# Postcranial anatomy of *Besanosaurus leptorhynchus* (Reptilia: Ichthyosauria) from the Middle Triassic Besano Formation of Monte San Giorgio (Italy/Switzerland), with implications for reconstructing the swimming styles of Triassic ichthyosaurs

**DOI:** 10.1186/s13358-024-00330-9

**Published:** 2024-09-10

**Authors:** Gabriele Bindellini, Andrzej S. Wolniewicz, Feiko Miedema, Cristiano Dal Sasso, Torsten M. Scheyer

**Affiliations:** 1 Dipartimento di Scienze della Terra “Ardito Desio”, Università degli Studi di Milano, Milan, Italy; 2 Dipartimento di Scienze della Terra, Sapienza Università di Roma, Rome, Italy; 3 Department of Earth Sciences, University of Cambridge, Cambridge, UK; 4 School of Resources and Environmental Engineering, Hefei University of Technology, Hefei, China; 5 Institute of Paleobiology, Polish Academy of Sciences, Warsaw, Poland; 6 Staatliches Museum Für Naturkunde Stuttgart, Stuttgart, Germany; 7 Sezione di Paleontologia dei Vertebrati, Museo di Storia Naturale di Milano, Milan, Italy; 8 Paläontologisches Institut, Universität Zürich, Zurich, Switzerland

**Keywords:** Ichthyosauria, Shastasauridae, Middle Triassic, Besano Formation, Monte San Giorgio, Postcranial anatomy, Osteology, Phylogeny, Swimming style, Marine reptiles

## Abstract

*Besanosaurus leptorhynchus* Dal Sasso & Pinna, 1996 was originally described on the basis of a single complete fossil specimen excavated near Besano (Italy). However, a recent taxonomic revision and re-examination of the cranial osteology allowed for the assignment of five additional specimens to the taxon. Here, we analyse, describe and discuss the postcranial anatomy of *Besanosaurus leptorhynchus* in detail. The size of the specimens examined herein ranged from slightly more than one meter to eight meters. Overall, several diagnostic character states for this taxon are proposed, demonstrating a mosaic of plesiomorphic and derived features. This is best exemplified by the limbs, which show very rounded elements in the forelimbs, and pedal phalanges with retained rudimentary shafts. We suggest that the widely spaced phalanges in the forefins of *Besanosaurus leptorhynchus* were embedded in a fibrocartilage-rich connective tissue, like in modern cetaceans. We also review the similarities of *Besanosaurus* with *Pessopteryx* and *Pessosaurus,* allowing us to conclude that *Besanosaurus* is not a junior synonym of either of the two taxa. Lastly, to test the swimming capabilities of *Besanosaurus leptorhynchus*, we expanded on a previously published study focussing on reconstructing the swimming styles of ichthyosaurs. *Besanosaurus* *leptorhynchus* was found to possess a peculiar locomotory mode, somewhat intermediate between anguilliform swimmers, such as *Cymbospondylus* and *Utatsusaurus*, and some shastasaur-grade (e.g., *Guizhouichthyosaurus*) and early-diverging euichthyosaurian (e.g., *Californosaurus*) ichthyosaurs. Based on our results, we furthermore suggest that mixosaurids acquired their characteristic body profile (dorsal fin and forefins that are distinctly enlarged compared to the hindfins) independently and convergently to the one that later appeared in Parvipelvia. Moreover, the different swimming styles inferred for *Cymbospondylus,* Mixosauridae*,* and *Besanosaurus* strengthen the earlier hypothesis of niche partitioning among these three distinct ichthyosaur taxa from the Besano Formation.

**Supplementary Information:**

The online version contains supplementary material available at 10.1186/s13358-024-00330-9.

## Introduction

Cymbospondylids and shastasaurs were important members of Triassic marine ecosystems, representing the earliest medium- to large-bodied ichthyosaurs, ranging in size from about 6 m to more than 20 m (Klein et al., 2020; Nicholls & Manabe, 2004; Sander et al., 2011, 2021, 2024). The monophyly of Shastasauridae (recovered by e.g., Huang et al., 2019; Ji et al., 2013, 2016; Jiang et al., 2016; Motani et al., 2017) has been questioned in the past, and the clade has been recovered as paraphyletic by several authors (e.g., Bindellini et al., 2021; Maisch & Matzke, 2000; Moon, 2019; Moon & Stubbs, 2020; Sander, 2000; Sander et al., 2011). In this paper, we consider the clade as paraphyletic and refer to the taxa previously included in Shastasauridae sensu Ji et al. (2016), i.e., *Shastasaurus*, *Besanosaurus*, *Guanlingsaurus* (junior synonym of *Shastasaurus* according to some authors; Sander et al., 2011; Moon et al., 2019), *Guizhouichthyosaurus*, *Shonisaurus*, and *‘Callawayia*' *wolonggangense* as shastasaur-grade ichthyosaurs (sensu Bindellini et al., 2021) and provide additional evidence in support to this designation.

**Fig. 1:**
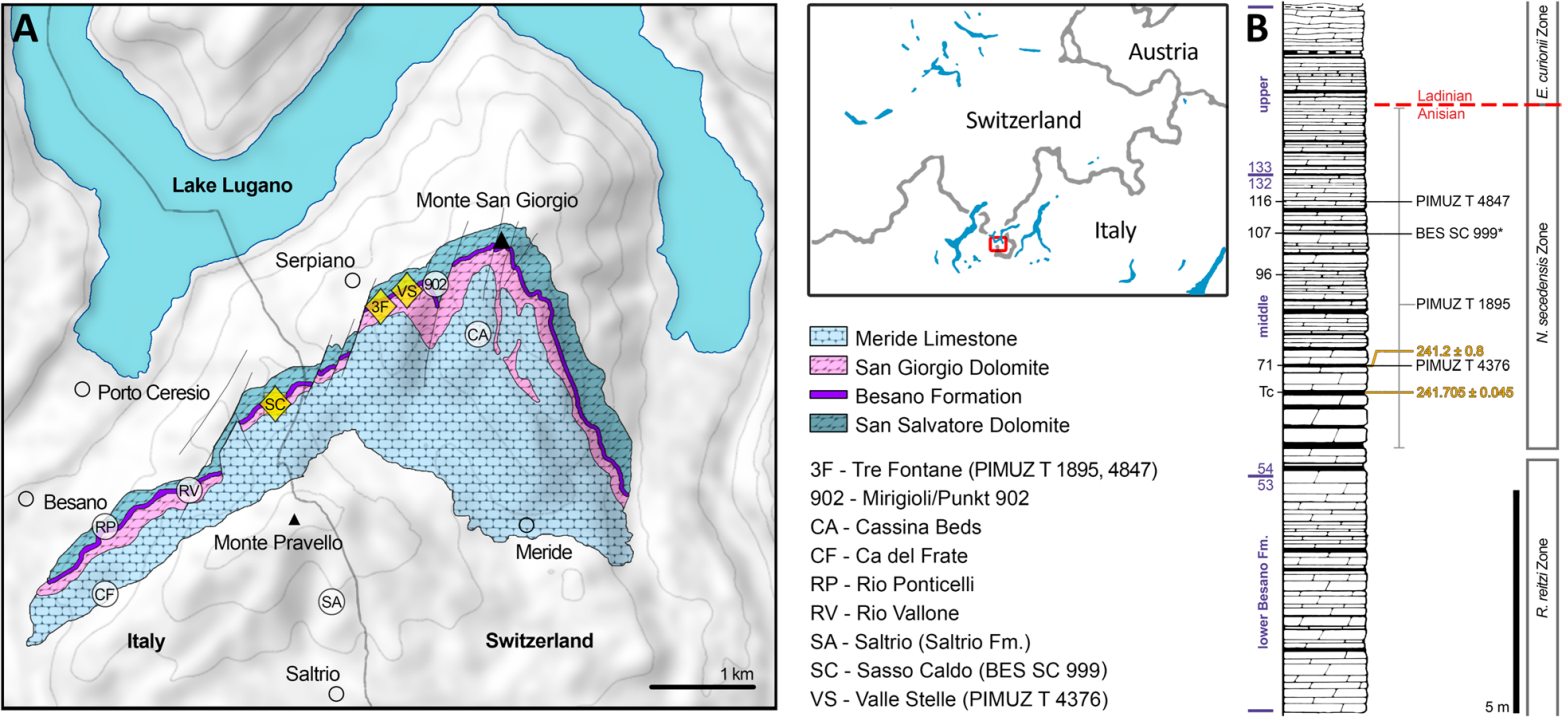
**A** Map of the Monte San Giorgio area showing the Middle Triassic carbonate succession, the major paleontological quarries in the area (white circles), and the sites of origin of the specimens described in this paper (yellow rhombuses). **B** Stratigraphic log of the Besano Formation at the Mirigioli/Punkt 902 outcrop, with the known stratigraphic positions of the specimens marked. The stratigraphic position of PIMUZ T 1895 and GPIT 1793/1 is uncertain, and thus expressed by a range line. Log modified from Brack et al. (2005); dating of layer 71 from Mundil et al. (1996); dating of Tc Tuffs (layers 66–68) from Wotzlaw et al. (2017)

Ichthyosaurs are greatly abundant fossil marine reptiles of the Monte San Giorgio UNESCO World Heritage Site (Canton Ticino, Switzerland, and Lombardy, Italy; Fig. 1), protected for its Middle Triassic marine fossil palaeobiodiversity (e.g., Rieppel, 2019). In the sedimentary succession of Monte San Giorgio, the Besano Formation (dated to the Anisian/Ladinian boundary) was found to be particularly rich in both vertebrate and invertebrate fauna. In this formation, three distinct clades belonging to Ichthyosauria have been recovered: Mixosauridae, *Cymbospondylus*, and the shastasaur *Besanosaurus.* The holotype of *Besanosaurus leptorhynchus* (BES SC 999; Dal Sasso & Pinna, 1996) was unearthed in 1993 in the Sasso Caldo quarry near Besano. Since its first description, it was found to show close affinity with shastasaur-grade ichthyosaurs, despite exhibiting several unique characters both in the skull and the postcranial skeleton. However, BES SC 999 was not the only shastasaur recovered from Monte San Giorgio/Besano. Three well-preserved specimens, two medium-sized (PIMUZ T 4376 and PIMUZ T 1895) and a large (PIMUZ T 4847) skeleton, were present in the collections of the Paläontologisches Institut und Museum der Universität Zürich (PIMUZ) since the late 1920s/early 1930s (PIMUZ records). These skeletons were briefly mentioned in 20th-century literature (Brinkmann, 1994, 1997; Cook, 1994; Kuhn-Schnyder, 1964; McGowan, 1976; Sander, 1989), and the medium-sized skeleton was seemingly under study in the 1990s (Cook, 1994; Dal Sasso & Pinna, 1996; Brinkmann, 1997), but a comprehensive osteological description was never published.

In this work, we revise the postcranial anatomy of *Besanosaurus leptorhynchus* and describe the postcranial skeletons of the above-mentioned shastasaurid specimens from the PIMUZ collections in detail for the first time. Furthermore, we comprehensively compare these specimens with other ichthyosaurs, make inferences on the swimming style of *Besanosaurus* and other Middle Triassic ichthyosaur taxa, infer the ossification patterns of some parts of the skeleton, and address taxonomic issues related with the Early–Middle Triassic ichthyosaurs *Pessopteryx* and *Pessosaurus* from Svalbard.

## Geological setting

The Besano Formation (also referred to as Grenzbitumenzone) crops out on Monte San Giorgio (Lombardy, Italy, and Canton Ticino, Switzerland; Fig. 1) and is one of the richest and most well-known sites of Middle Triassic marine palaeobiodiversity (e.g., Benton et al., 2013; Rieppel, 2019). Four different formations form the Middle Triassic carbonate succession of Monte San Giorgio; they were deposited on the western margin of the Neo-Tethys in a carbonate platform sedimentological context (Bernasconi, 1994; Etter, 2002; Furrer, 1995; Röhl et al., 2001; Stockar et al., 2012). The formations are the San Salvatore Dolomite, the Besano Formation, the San Giorgio Dolomite, and the Meride Limestone (Fig. 1). During the Anisian, after the deposition of the Salvatore Dolomite, the development of a 30–130 m deep and approximately 20 km wide basin resulted in the deposition of the Besano Formation (e.g., Bernasconi, 1991, 1994; Bernasconi & Riva, 1993; Furrer, 1995). It is 5 to 16 m thick and consists of an alternation of black shales and variably laminated, organic-rich dolomitic layers. Subordinate cineritic tuffs were used to date the formation to the Anisian/Ladinian boundary (Brack & Rieber, 1986, 1993; Brack et al., 2005; Mundil et al., 1996; Wotzlaw et al., 2017). The formation is divided into three portions (Röhl et al., 2001): the upper and lower portions of the Besano Fm. represent a restricted, shallow, inter-to-subtidal carbonate platform rich in nearshore vertebrates, whereas the middle part is a slightly deeper intraplatform basin, from which a great number of ichthyosaurian and other pelagic vertebrate remains have been recovered (e.g., Bürgin et al., 1989; Dal Sasso & Pinna, 1996; Brinkmann, 1997; Maisch & Matzke, 1998; Furrer, 2003; Renesto et al., 2020; Bindellini et al., 2021; Bindellini & Dal Sasso, 2022; Miedema et al., 2023; Viaretti et al., 2020, 2023). At the Sasso Caldo site near Besano, only the middle and the upper portions of the Besano Formation crop out. Recent biozonation of the Sasso Caldo site (Bindellini, 2022; Bindellini et al., 2019) allowed a confident correlation of this stratigraphic section with the Swiss localities (Brack & Rieber, 1993; Brack et al., 2005; Rieber, 1973).

The four specimens referred to *Besanosaurus leptorhynchus* described in this work originate from the middle portion of the Besano Formation and are dated to the *N. secedensis* Zone. The holotype of *Besanosaurus leptorhynchus* (BES SC 999) was collected at the Sasso Caldo site from the equivalent of layer 107 of the Besano Formation type section (Bindellini et al., 2021; Dal Sasso & Pinna, 1996). PIMUZ T 1895 and PIMUZ T 4847 come from the Cava Tre Fontane site. The latter was recovered from layer 116, whereas the exact horizon of origin of the former is unknown. PIMUZ T 4376 comes from the Valle Stelle site and was collected from layer 71 (Bindellini et al., 2021; Furrer, 2024; Fig. 1).

## Material and methods

### Material

**Fig. 2:**
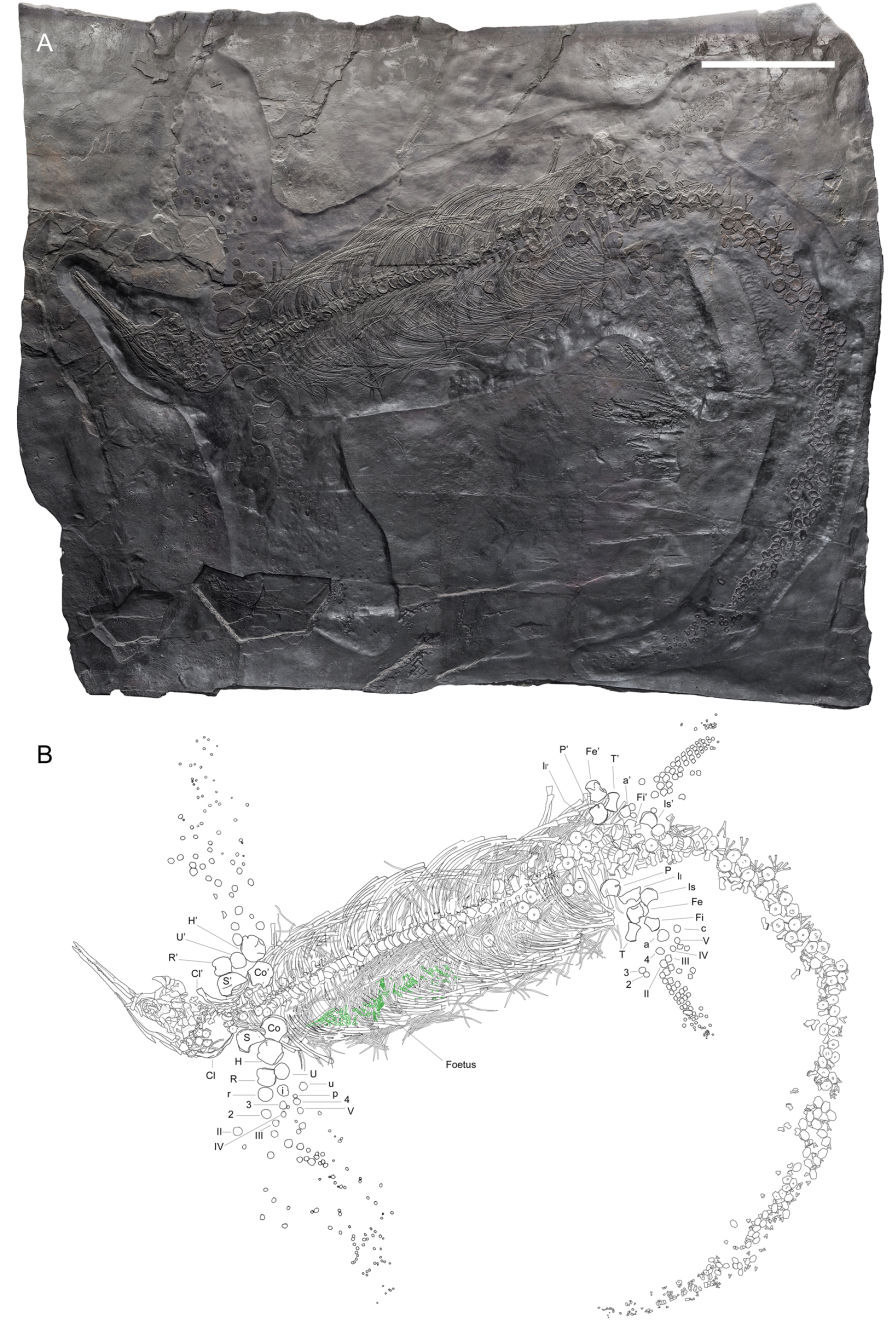
**A** Cast of BES SC 999, the holotype of *Besanosaurus leptorhynchus* and (**B**) interpretative drawing (modified from Dal Sasso & Pinna, 1996). Foetal remains are highlighted with green lines. *a* astragalus, *c* calcaneum, *Cl* clavicle, *Co* coracoid, *Fe* femur, *Fi* Fibula, *H* humerus, *i* intermedium, *Il* Ilium, *Is* Ischium, *P* pubis, *p* pisiform, *R* radius, *r* radiale, *S* scapula, *T* Tibia, *U* Ulna, *u* ulnare; 2, 3, and 4, distal carpals and tarsals; II, III, IV, and V, metacarpals and metatarsals. The apostrophe (‘) indicates left elements. Scale bar represents 50 cm

**Fig. 3:**
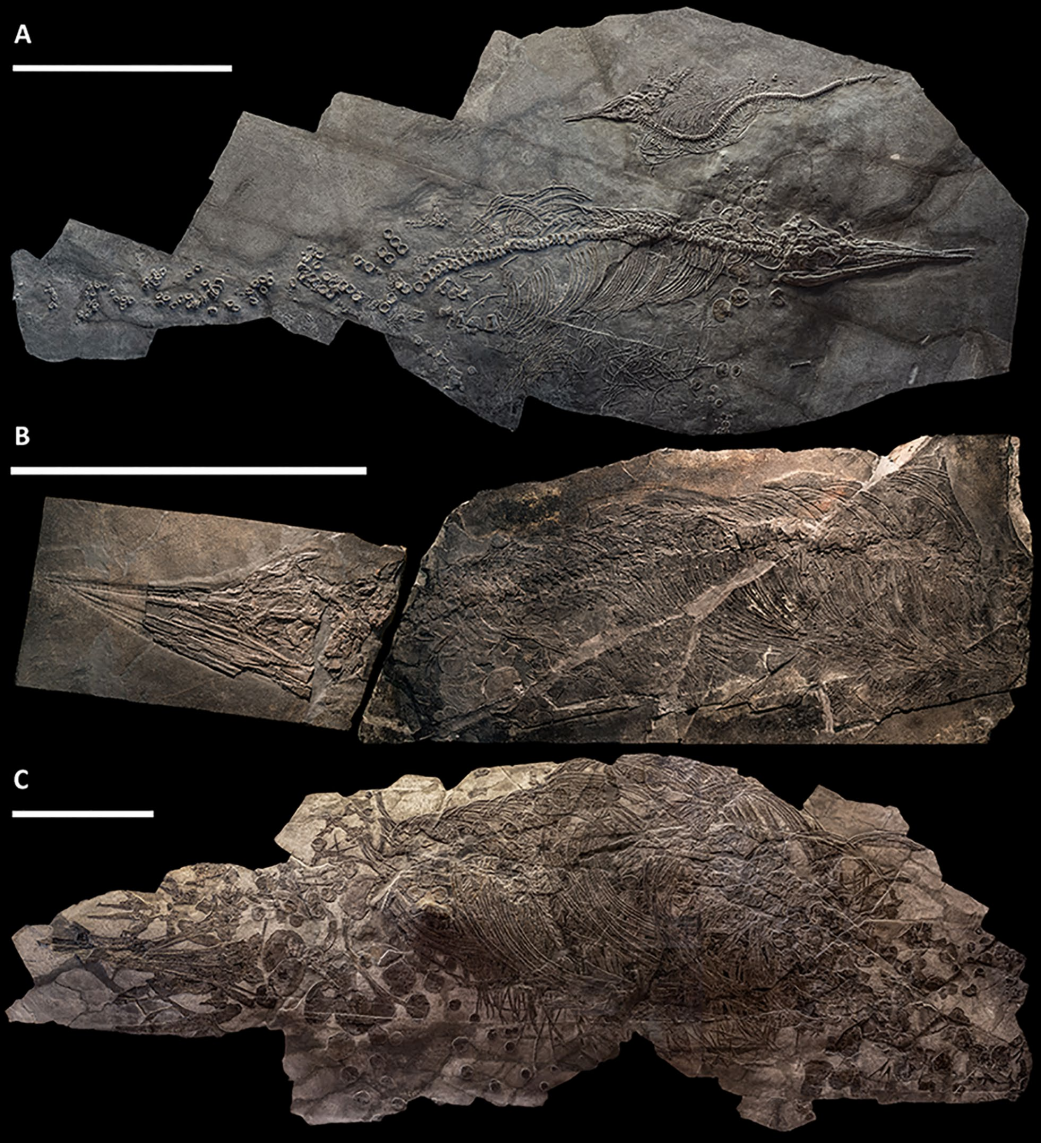
Three additional skeletons referred to *Besanosaurus leptorhynchus*. **A** PIMUZ T 4376, the smallest individual, an almost complete specimen with disarticulated tail and missing some of the elements from the fins; **B** PIMUZ T 1895, a smaller individual missing most of the fins, sacrals and postsacral elements; a portion of the tail of PIMUZ T 1895 is preserved in an additional block (Fig. 5A); **C** PIMUZ T 4847, a large adult missing most of the fins, sacrals and postsacral elements. Scale bars represent 50 cm

All studied specimens come from the Monte San Giorgio area (Fig. 1), where fossils lay in a single bedding plane and are compressed by diagenetic alteration and fossilisation (Figs. 2 and 3). Disarticulation is more common in the forefins than in the hindfins, and in the postsacral rather than presacral axial skeleton (BES SC 999, PIMUZ T 4376, PIMUZ T 4847). One specimen, the holotype, BES SC 999, contains foetal and soft tissue remains, whereas the largest specimen, PIMUZ T 4847, contains a large pyritised mineral nodule in the cranial half of the thoracic region, possibly related to visceral soft tissue. All specimens underwent extreme taphonomical compression, and therefore bones are often preserved in a very thin layer, sometimes as thin as a few millimetres. PIMUZ T 4376 represents a partial exception to this rule since some anatomical features are preserved more three-dimensionally in this specimen. The extreme taphonomic compression greatly distorted some of the preserved morphological features, especially in the limbs and girdles, necessitating caution in interpreting the actual anatomy of the specimens (see also the skull description, Bindellini et al., 2021).

**Fig. 4:**
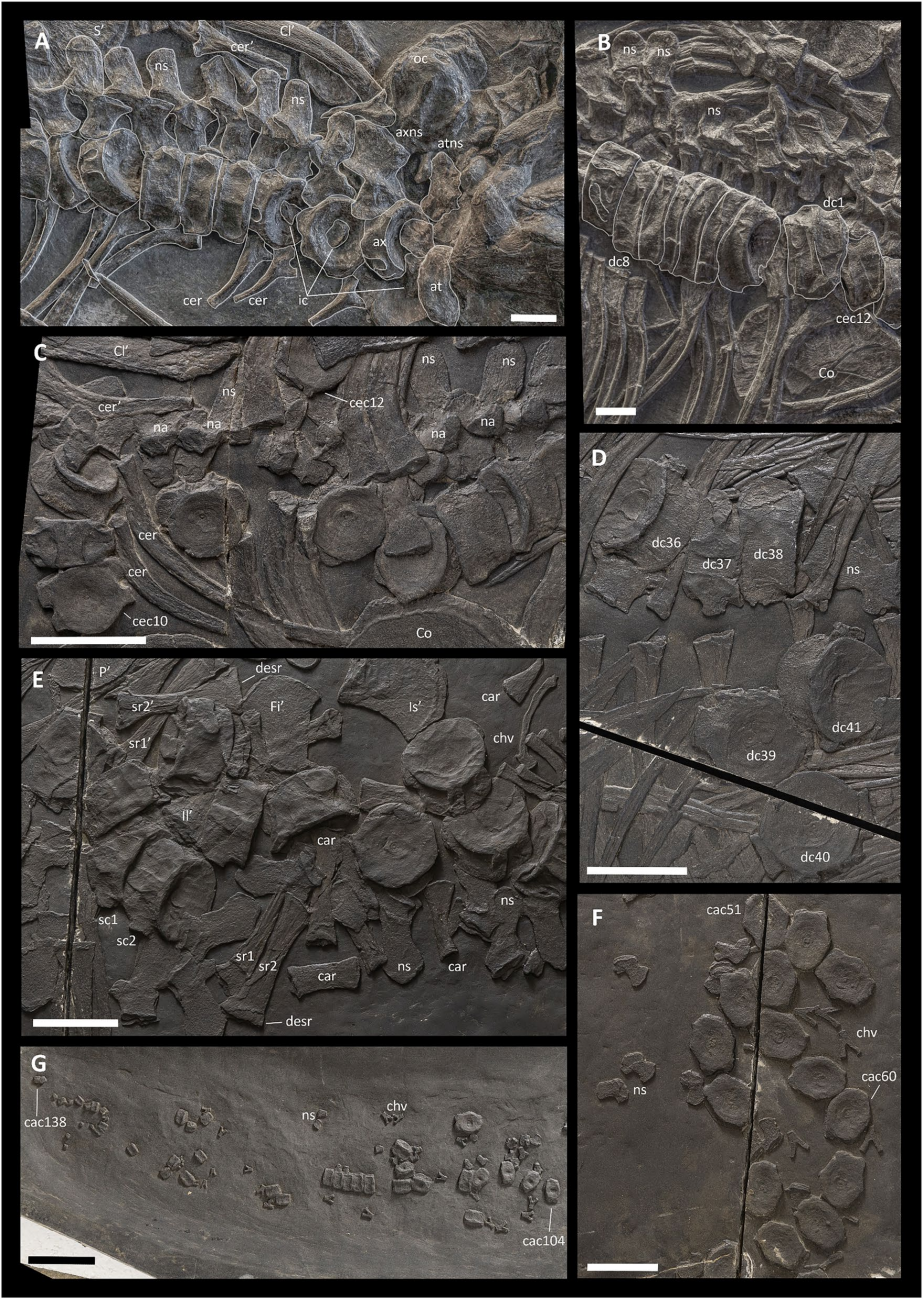
Selected elements of the axial skeleton of *Besanosaurus leptorhynchus*. **A**, **B** portions of the axial skeleton of PIMUZ T 4376, including the cervical series and the anteriormost portion of the dorsal region; **C** BES SC 999, posteriormost cervical and anteriormost dorsal vertebrae; **D** BES SC 999, vertebrae belonging to the posterior half of the presacral dorsal series; **E** BES SC 999, sacral region and anteriormost portion of the caudal series; **F** BES SC 999, caudal vertebrae around the inferred position of the tailbend; **G** BES SC 999, caudalmost region of the tail. *at* atlas, *ax* axis, *cac* caudal centrum, *car* caudal rib, *cec* cervical centrum, *cer* cervical rib, *chv* chevron, *Cl* clavicle, *Co* coracoid, *dc* dorsal centrum, *desr* distal end of sacral rib, *Fi* fibula, *ic* intercentra, *Il* ilium, *Is* ischium, *na* neural arch, *ns* neural spine, *oc* occipital condyle, *P* pubis, *S* scapula, *sc* sacral centrum, *sr* sacral rib. Numbers indicate the relative position of the centra within each of the vertebral column sections (ce, cervical; d, dorsal; ca, caudal). The apostrophe (‘) indicates a left element. Scale bars represent 1 cm in **A** and **B**, 5 cm in **C**–**F**

The studied specimens can be ordered according to increasing body size as follows:

PIMUZ T 4376—the smallest specimen (Figs. 3, 4, 6). It measures 2.12 m from the tip of the rostrum to the last preserved caudal vertebra (the distalmost caudals are missing).

**Fig. 5:**
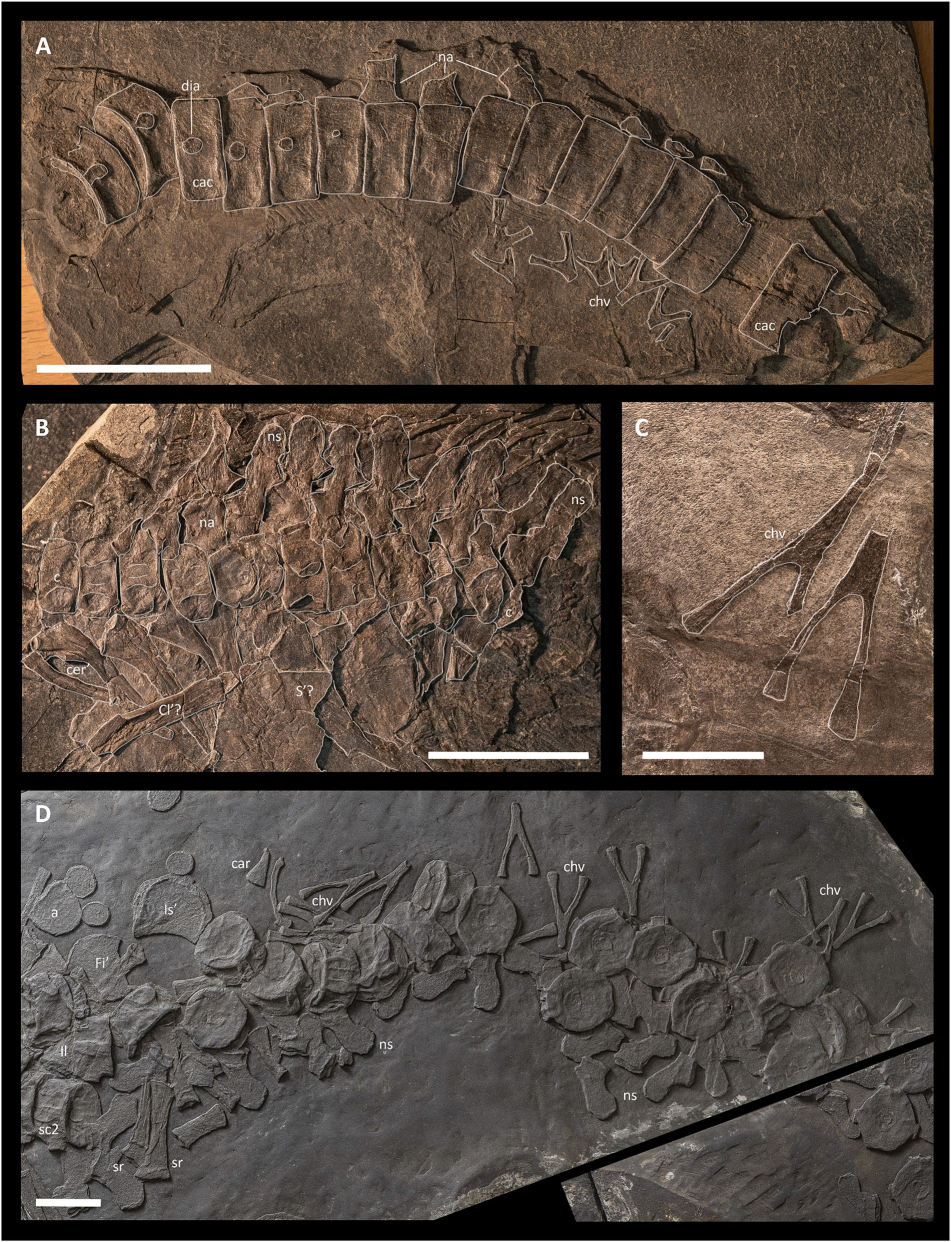
Selected elements of the axial skeleton of *Besanosaurus leptorhynchus*. **A**, **B** PIMUZ T 1895, tailbend region and anteriormost region of the presacral vertebral series, respectively; **C** two chevrons belonging to PIMUZ T 4847; **D** BES SC 999, anteriormost portion of the tail. *a* astragalus, *c* centrum, *cac* caudal centrum, *car* caudal rib, *chv* chevron, *Cl* clavicle, *dia* diapophysis, *Fi* fibula, *Il* ilium, *Is* ischium, *na* neural arch, *ns* neural spine, *S* scapula, *sc* sacral centrum, *sr* sacral rib. Numbers indicate the position of the centra within each of the vertebral column sections (ce, cervical; d, dorsal; ca, caudal). The apostrophe (‘) always indicates a left element. Scale bars represent 5 cm

PIMUZ T 1895—the specimen is incomplete (lacking most of the tail and the limbs, except for the tailbend) but mostly articulated (Figs. 3, 5). The preserved presacral length is around 1.40 m. The preserved elements suggest that the absolute size of this specimen was intermediate between that of PIMUZ T 4376 and BES SC 999.

**Fig. 6:**
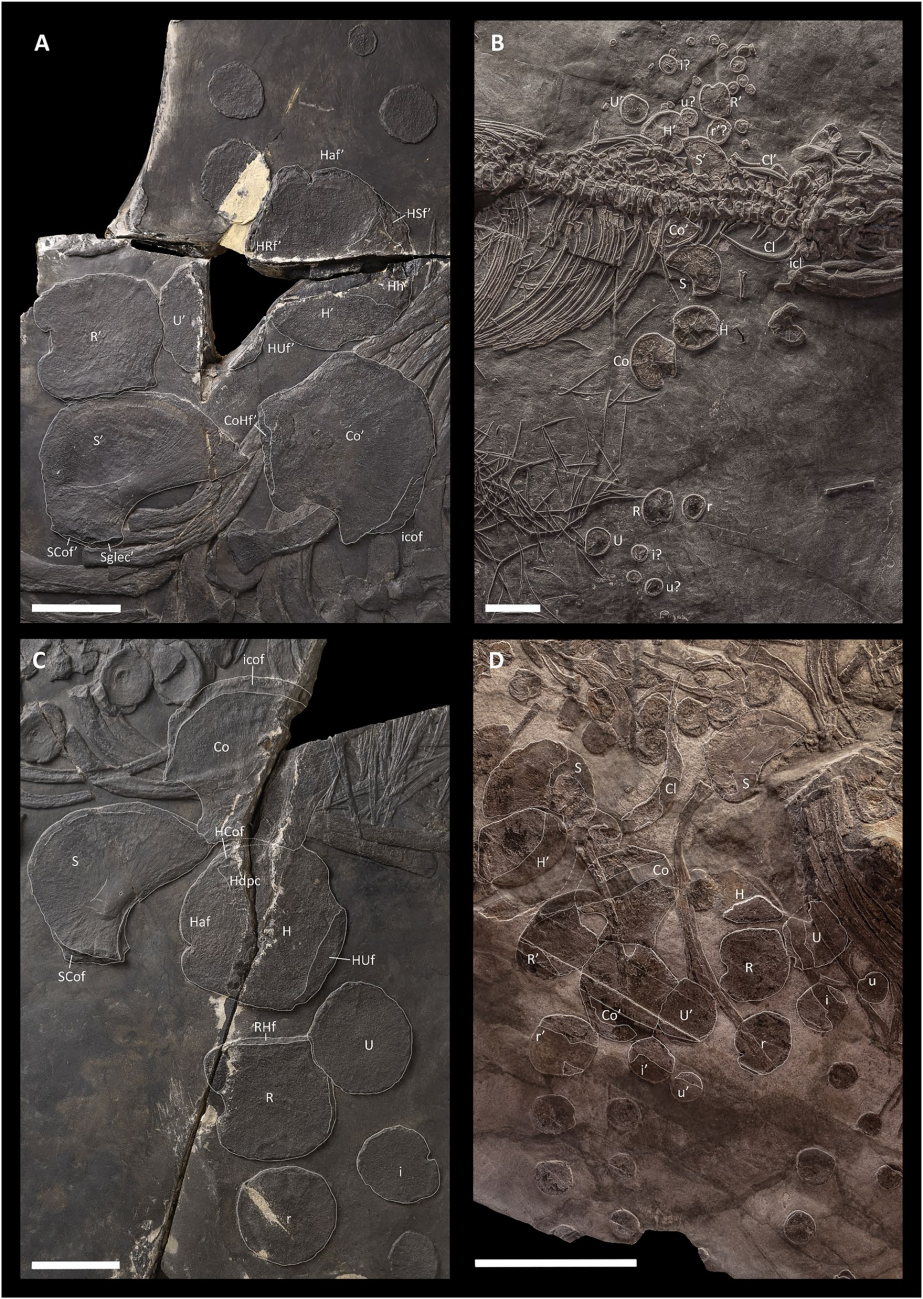
The left (**A**) and right (**C**) forefin and shoulder girdle elements of BES SC 999, and forefin and shoulder girdle elements of PIMUZ T 4376 (**B**) and PIMUZ T 4847 (D). *Cl* clavicle, *Co* coracoid, *CoHf* coracoid facet for the humerus, *H* humerus, *HSf* humerus facet for the scapula, *Haf* anterior flange of the humerus, *HCof* humerus facet for the coracoid, *Hdpc* deltopectoral crest of the humerus, *Hh* humerus head, *HRf* humerus facet for the radius, *HUf* humerus facet for the ulna, *i* intermedium, *icof* intercoracoidal facet, *r* radiale, *R* radius, *RHf* radial facet for the humerus, *S* scapula, *Sglec* glenoid contribution of the scapula, *SCof* scapular facet for the coracoid, *U* ulna. The apostrophe (‘) always indicates a left element. In **A**, **B**, and **C** scale bars represent 5 cm; in **D** it represents 25 cm

BES SC 999—the holotype of *Besanosaurus leptorhynchus*, a pregnant female (Figs. 2, 4, 5, 6). It measures 5.07 m from the tip of the rostrum to the last caudal vertebra. The skeleton is virtually complete and exposed in ventral view.

PIMUZ T 4847—the largest specimen described in this work (Figs. 3, 6). It has a presacral length of 3.28 m but lacks most of the postsacral skeleton and most of the limb bones. In life, this individual likely reached a length of about 8 m (Bindellini et al., 2021).

In addition, two complete and articulated specimens referred to *Mixosaurus cornalianus* (BES SC 1000 and BES SC 1001) (Renesto et al., 2020) were personally examined during the preparation of this study and used solely for comparative purposes.

### Methods

X-ray computed tomography (CT) was performed on BES SC 999, the holotype of *Besanosaurus leptorhynchus*, with a Siemens Somatom Definition Dual Source CT Scanner at the Radiology Department of the Fondazione IRCCS “Cà Granda” Ospedale Maggiore Policlinico di Milano. The best CT imaging was obtained with a bone algorithm on transverse (axial) slices with a voltage of 140 kV, a current of 180–270 mA, and a slice thickness of 0.3 mm (Crasti, 2019). Data were exported in DICOM format using eFilm (v. 1.5.3; Merge eFilm, Toronto, ON, Canada). Analysis and post-processing were performed with RadiAnt, 3DimViewer, and Synedra View Personal. Multiplanar reconstructions (MPR) and volume rendering reconstructions (VR) allowed us to inspect the bones hidden from external view by other bones embedded within the matrix, otherwise impossible to study without damaging the fossil.

Photogrammetry was used to better inspect the postcranial anatomy of specimens BES SC 999 and PIMUZ T 4376. Photos of all studied specimens were taken with a Nikon D3500 camera using the RAW file format. Figures included in the manuscript are slightly post-produced versions of these RAW files to enhance highlights and shadows.

To assess the phylogenetic position of *Besanosaurus leptorhynchus*, a phylogenetic analysis was performed in TNT 1.5 (Goloboff & Catalano, 2016). The analysis was based on that presented by Bindellini et al. (2021), with postcranial character scores for *Besanosaurus leptorhynchus* updated on the basis of this study.

To investigate the swimming mode of *Besanosaurus leptorhynchus*, we plotted 27 additional taxa (16 ichthyopterygians and 11 fishes) onto a body shape diagram originally produced by Motani et al. (1996) (see also Motani, 2005, 2008; Lindgren et al., 2013). This procedure allowed for the inclusion of a broader representation of taxa in terms of phylogenetic diversity and swimming styles than the original analysis of Motani et al. (1996). The “caudal fin H/L ratio” (caudal fluke height divided by fluke length) is plotted on the x-axis and the “fineness ratio” (body height—excluding the dorsal fin—divided by prefluke length) is plotted on the y-axis. The fish taxa included in the analysis represent different swimming modes, likely also exhibited by ichthyopterygians (anguilliform, subcarangiform, carangiform, and thunniform) (McGowan, 1992; Motani, 2008; Motani et al., 1996). We decided to add 10 taxa belonging to Scombridae, to show where extant taxa of thunniform swimmers plot, since this swimming mode was underrepresented in the original analysis of Motani et al. (1996). Silhouettes of both ichthyosaurs and fishes, along with their sources, as well as the measurements obtained from them, are included in Fig. S6 and Tab. S1. Since the height of the dorsal lobe of the caudal fluke is not known in *Californosaurus*, *Temnodontosaurus*, and *Eurhinosaurus*, we plotted three different possible reconstructions for these genera: one in which the dorsal lobe of the caudal fluke is as reconstructed in Fig. S6, one in which it is 50% the height of the ventral lobe, and one in which it is 25% of the height.

## Systematic palaeontology

ICHTHYOPTERYGIA Owen, 1859

ICHTHYOSAURIA Blainville, 1835

MERRIAMOSAURIA Motani, 1999

*BESANOSAURUS* Dal Sasso & Pinna, 1996

*Besanosaurus leptorhynchus* Dal Sasso & Pinna, 1996

**Type and only species** *Besanosaurus leptorhynchus* Dal Sasso & Pinna, 1996.

**Type specimen** Complete, mostly articulated skeleton (Fig. 2), listed as BES SC 999 in the catalogue of the MSNM (BES SC is an acronym for the Besano Sasso Caldo quarry) and coded as 20.S288-2.2 in the Inventario Patrimoniale dello Stato (Italian State Heritage Database).

**Type locality** Sasso Caldo site, Besano, Varese Province, NW Lombardy, N Italy. Geographical coordinates: 45°54′03.7ʺN 8°55′10.6ʺE, elev. 650 m.

**Type horizon and distribution** Middle portion of the Besano Formation (sensu Bindellini et al., 2019), uppermost Anisian (*N. secedensis* Zone sensu Brack et al., 2005), Middle Triassic.

**Referred material** PIMUZ T 4376 (complete, mostly articulated skeleton with the best-preserved skull of the taxon; Fig. 3A), PIMUZ T 1895 (incomplete, mostly articulated skeleton with a well-preserved skull; Fig. 3B), PIMUZ T 4847 (incomplete, mostly disarticulated skeleton with a disarticulated skull; Fig. 3C), GPIT 1793/1 (disarticulated skull, the holotype of *Mikadocephalus gracilirostris* Maisch & Matzke, 1997), BES SC 1016 (incomplete, mostly disarticulated skull; the specimen is coded as 20.S288-2.6 in the Inventario Patrimoniale dello Stato (Italian State Heritage Database).

**Emended diagnosis** Large ichthyosaur (estimated adult body length ~ 8 m) with one possible autapomorphy—a caudoventral exposure of the postorbital in the temporal region—and the following combination of character states: extremely long, slender, and gracile snout; frontal rostrocaudally elongate and relatively flat; frontal participation in the temporal fossa (= anterior terrace; Motani, 1999) but not in the temporal fenestra; L-shaped jugal; ‘triangular process’ on the medioventral border of the quadrate; prominent coronoid (preglenoid) process of the surangular, distinctly raised above its dorsal margin; tiny, conical teeth with a coarsely-striated crown surface and deeply striated roots; mesial maxillary teeth set in sockets; distal maxillary teeth set in a groove shorter than half of the rostral ramus of the maxilla; 61 presacral vertebrae, at least two sacral vertebrae, and at least 138 caudal vertebrae; tailbend forming an angle of ~ 30°; wedge-shaped caudal centra located between the 56–60th position in the caudal series, possessing small and rounded articular surfaces for possibly unossified ribs; caudal series comprising 55% of the length of the axial skeleton in adults; rounded humerus; rounded manual phalanges; pedal phalanges retaining reduced shafts in adults; obturator foramen with a sub-oval outline, open in juveniles and almost entirely enclosed within the pubis in adults.

**Remarks** Several additional specimens at the PIMUZ are potentially referable to *Besanosaurus* and are treated as aff. *Besanosaurus* herein. Previously, these specimens were preliminarily identified in the field as *Mixosaurus* (except PIMUZ T 2027, which was labelled as Shastasauridae indet.) and include: PIMUZ T 188, two isolated paddle elements preserved on the surface of one matrix slab; PIMUZ T 190, isolated and badly preserved paddle elements on two matrix slabs; PIMUZ T 1935, jaw fragments on one slab; PIMUZ T 1973, incomplete skeletal remains on nine (potentially ten if one unnumbered slab is counted as well) matrix slabs; PIMUZ T 2010, skull remains on two slabs; PIMUZ T 2013, large skeletal remains including trunk and paddle remains on three slabs (incl. T 2015, which was identified as a counterslab to one of the two slabs of T 2013); PIMUZ T 2027, vertebral elements (several sectioned histologically) on one slab; PIMUZ T 2141, vertebrae and a girdle element on one slab; PIMUZ T 2144, skull and lower jaw remains; PIMUZ T 2259, jaw remains on two slabs; PIMUZ T 2359, articulated vertebrae with chevron bones on one slab; and PIMUZ T 2759, large jaw fragments on one slab.

## Description

### Axial skeleton

#### Vertebrae

We recognise 12 cervical centra, 49 dorsal centra, at least two sacral centra, and at least 138 caudal centra (201 vertebrae in total) in the axial skeleton of the holotype of *Besanosaurus leptorhynchus* (BES SC 999). The overall count of presacral vertebrae is at least 61. In PIMUZ T 4376, the caudalmost portion of the tail is not preserved, missing about 30 vertebrae from the tip of the tail. The preflexural and postflexural counts are 57 and 81, respectively. Apical wedge-shaped caudal centra are located between the 56–60th position in the caudal series. Cervical, dorsal, and proximal caudal centra are generally subcircular in outline in articular (anterior/posterior) view; mid-caudal centra become ovoid in outline and mediolaterally compressed (Dal Sasso & Pinna, 1996: fig. 13). All centra are amphicoelous and possess the typical ichthyosaurian hourglass-shaped sagittal cross-section.

*Cervical centra*—In PIMUZ T 4376, the specimen in which the neck region is best preserved, 12 cervical centra are present (Fig. 4A–C). These are exposed in right lateroventral view and show facets for dichocephalous ribs; therefore, we regard them as cervical vertebrae (following Sander, 1989). BES SC 999 also shows 12 cervical centra possessing both a diapophysis and a parapophysis (Fig. 4C). Except for the presence of diapophyses and parapophyses, the cervical centra are generally circular along the transverse plane, but the cranial-most centra show a triangular ventral outline that becomes rounder towards the dorsal region. The height/length ratio of the cervical vertebrae is ~ 1.5, which is much greater than in *Grippia longirostris* (~ 1.24; Ekeheien et al., 2018), but slightly lower than in *Cymbospondylus petrinus* and *Shastasaurus osmonti* (both ~ 1.6; Merriam, 1902, 1908).

The atlantal and axial pleurocentra are separate both in PIMUZ T 4376 and BES SC 999, although the atlantal pleurocentrum could only be identified in PIMUZ T 4376 (Fig. 4A). The atlas in PIMUZ T 4376 shows a concave anterior articular facet, like in mixosaurids, shastasaurids and more derived ichthyosaurs, but unlike in *Chaohusaurus* and *Cymbospondylus*, in which it is convex (Huang et al., 2019; McGowan & Motani, 2003; Merriam, 1902, 1908). The axial pleurocentrum does not show distinctive differences when compared to the more posterior cervical vertebrae. Three potential intercentra were also identified in PIMUZ T 4376 (Fig. 4A). Diapophyses and parapophyses are always present on the cervical centra; the diapophyses project dorsolaterally and the parapophyses project ventrolaterally (more markedly in the large specimens than in the small ones). The diapophyses increase in size posteriorly towards the dorsal region, gradually transitioning from a round to a reniform outline, whereas the parapophyses decrease in size maintaining a rounded outline. Anteriorly, the diapophyses and parapophyses are separated from each other, the dorsoventral distance between them decreases posteriorly, and the parapophyses are no longer present on the 13th centrum, in which only an enlarged diapophysis is visible (Fig. 4C). The diapophyses always contact the facets for the neural arch (Fig. 4A–C).

In BES SC 999, the 12th centrum possesses a parapophysis that is extremely reduced in size (Fig. 4C). A similar condition was described for *Guizhouichthyosaurus tangae* (Shang & Li, 2009) and *Shastasaurus pacificus* (Merriam, 1902: pl. 8, fig. 3). In *Shonisaurus sikanniensis* there is a parapophysis on the first 10 and in the 12th presacral centra, whereas the 11th centrum only shows a single rib facet (Nicholls & Manabe, 2004). Around 10 cervical centra were identified in *Guanlingsaurus liangae* (Ji et al., 2013), whereas a parapophysis is visible up until the 9th centrum in the cervical series of *Shonisaurus popularis* (Camp, 1980: fig. 28). The number of cervical vertebrae seems to be variable in *Cymbospondylus*. Sander (1989) identified six cervical vertebrae in *Cymbospondylus buchseri*, whereas Merriam (1908: fig. 58) reported the presence of a parapophysis on the first 12 centra of *Cymbospondylus petrinus*. Furthermore, Fröbisch et al. (2006) noted the presence of a tiny parapophysis on the 8th presacral centrum of *Cymbospondylus nichollsi* and suggested the 9th to have only a rudimentary parapophysis.

*Dorsal centra*—Dorsal centra (i.e., non-cervical presacral centra) are generally circular in articular view, showing a regular and constant morphology along the vertebral column. The height/length ratio of the dorsal centra slightly increases posteriorly along the trunk region (~ 1.5 for the cranialmost dorsal centra; ~ 1.75 for the caudalmost dorsal centra), and the overall size of the centra increases from the neck towards the pelvis (in the holotype, the height of the 10th cervical centrum equals 31 mm and the height of the 46th dorsal centrum equals 49 mm). For comparison, the height/length ratio of the dorsal centra in *Cymbospondylus buchseri* (Sander, 1989) and *Shastasaurus osmonti* (Merriam, 1902, 1908) is much greater than in *Besanosaurus leptorhynchus* with a mean value of ~ 2.

On the lateral side, the dorsal centra have only one articular facet for holocephalous ribs. The diapophysis is dorsoventrally long on all dorsal centra, has a concave cranial margin, and shows a slight constriction in the middle (Fig. 4D). It is generally confluent with the anterior articular surface of the centrum, although never truncated by it (Fig. 4D). This condition is more similar to *Shastasaurus* and *Californosaurus* (Merriam, 1902, 1908) than to *Cymbospondylus* (Sander, 1989, 1992), in which the diapophysis is visibly truncated by the anterior articular facet. The diapophysis contacts the facet for the neural arch up until at least the 37th or 38th dorsal centrum (the 49th and 50th centrum in general, respectively; Fig. 4D). In comparison, in *Cymbospondylus buchseri* the connection between the rib facet and the neural arch is lost at the 29th dorsal centrum (35th centrum overall; Sander, 1989), in *Cymbospondylus petrinus* it is lost between the 24th and 26th dorsal centrum (36th–38th centrum in general; Merriam, 1908), in *Californosaurus perrini* it is lost in the 27th dorsal centrum (Merriam, 1902), and in *Shonisaurus popularis* it is already lost anterior to the 23rd dorsal centrum (Camp, 1980). The dorsal centra of *Besanosaurus leptorhynchus* differ greatly from those of *Mixosaurus*: in the latter, they are relatively longer (height/length ratio of a mid-dorsal centrum in BES SC 1000 is 1.45) and two rib facets are visible on the lateral side of the centra close to the pelvic region (e.g., Brinkman, 2004; Renesto et al., 2020). In lateral view, the dorsal centra of *Besanosaurus leptorhynchus* and *Cymbospondylus* are very similar. However, unlike in *Cymbospondylus buchseri*, the posterior dorsal vertebrae of *Besanosaurus leptorhynchus*, except for the last four caudalmost ones, possess a diapophysis located around or above the mid-height of the centrum that never shows the anteroventral extension visible in the dorsal centra of the caudal half of the trunk in *Cymbospondylus buchseri* (Sander, 1989). In addition, the typical triangular shape of the posterior dorsals in craniocaudal view of *Cymbospondylus petrinus* (Merriam, 1908) and likely *Cymbospondylus buchseri* (Sander, 1989) differs from *Besanosaurus leptorhynchus*, in which the centra are sub-pentagonal in shape (Dal Sasso & Pinna, 1996). The dorsal centra of *Besanosaurus leptorhynchus* are also similar to those of *Shastasaurus osmonti* (Merriam, 1902) in that they are sub-circular in outline in articular view. However, in *Besanosaurus leptorhynchus* the neural arch facets are raised markedly above the dorsal surface of the centrum in posterior dorsals, in contrast to *Shastasaurus osmonti*, in which they are proportionally much lower. In addition, the diapophysis almost never contacts the anterior articular surface of the dorsal centra in *Shastasaurus osmonti* and is dorsoventrally much taller than in *Besanosaurus leptorhynchus*.

*Aberrant dorsal centra*—In PIMUZ T 4376, the first eight dorsal centra (the 13–20 centra overall; Fig. 4B) are remarkably narrow in lateral view in comparison with the preceding and following centra. Each of these centra measures about half of a normal centrum in length. We rule out the possibility that taphonomical compression selectively and plastically reduced the craniocaudal length of these eight centra since the associated elements (ribs and neural arches) maintain typical proportions. On the other hand, these centra are preserved packed closely to each other. To explain this particular condition, we tentatively suggest the existence of a developmental abnormality that affected the craniocaudal length of centra 13–20 in PIMUZ T 4376.

*Sacral centra*—We distinguish at least two sacral vertebrae in BES SC 999, identified by the presence of at least two large, disarticulated rib pairs with clearly expanded distal ends, that we recognise as sacral ribs (Fig. 4E). Two sacral centra were also reported in *Shonisaurus popularis* (Camp, 1980) and *Guizhouichthyosaurus tangae* (Shang & Li, 2009). Sacral centra are also preserved in PIMUZ T 4376 but are not visible in PIMUZ T 1895 and T 4847. The sacral centra in *Besanosaurus leptorhynchus* occupy the 62nd and 63rd position along the vertebral column (and possibly two further positions caudally), so that the presacral vertebral count can be determined as 61. The sacral centra do not show diagnostic characters that can clearly differentiate them from the caudalmost dorsal centra and the cranialmost caudal centra. A gradual, slight increase in size in the caudal direction of the precaudal vertebrae makes the two sacral centra the largest in the entire axial skeleton (63rd centrum measures 52 mm in mediolateral width and 25 mm in craniocaudal length in BES SC 999).

In lateral view, both sacral vertebrae show a dorsoventrally elongated diapophysis that is reniform in outline and facets for neural arches that are enlarged lateromedially in the middle.

*Caudal centra*—BES SC 999 is the only specimen that possesses a complete, partially articulated tail (Figs. 2, 4, and 5). In this specimen, the caudal series (possessing at least 138 vertebrae) measures ~ 280 cm (measured along the vertebral column, without taking the tailbend into account), which is ~ 1.2 times the length of the rest of the body and accounts for 55.3% of the entire length of the axial skeleton. In the holotype, the tailbend is inferred to be present 155 cm caudally to the sacrum, based on the position of the tailbend preserved in PIMUZ T 1895 (see below). PIMUZ T 4376 also preserves an almost complete tail measuring ~ 104 cm, albeit it is mostly disarticulated and comprises at least 105 caudal centra, missing only the distalmost ones.

The size of the caudal centra decreases immediately posterior to the sacrum. Moreover, the postsacral centra gradually become more and more mediolaterally compressed. Centra that are clearly dorsoventrally taller than mediolaterally wide are visible posterior to the 30th caudal centrum of BES SC 999 (Figs. 4F, and 5D). From the cranialmost to the caudalmost position, the height/length ratio of caudal preflexural centra gradually changes from ~ 1.8 to ~ 2.8, whereas in the caudalmost postflexural preserved centra the ratio is ~ 1.65.

Wedge-shaped centra are visible on one of the slabs comprising PIMUZ T 1895, where at least five centra with a trapezoidal outline in lateral view can be recognised (Fig. 5A). The neural spines corresponding to these centra are shorter than the preflexural neural spines and oriented vertically (Fig. 4F). Neural spines positioned caudally to the tailbend become even shorter and cranially oriented. The tailbend in PIMUZ T 1895 possesses an angle of ~ 30°. In comparison, the tailbend reported in *Guanlingsaurus liangae* formed an angle of approx. 15° and the tailbend in *Shonisaurus popularis* formed an angle of only about 4° (Ji et al., 2011; McGowan & Motani, 1999). A slight tailbend was also reported for *Cymbospondylus petrinus*, but the angle it formed is not clear (Holger & Kosch, 1993).

In BES SC 999, the cranialmost 4 or 5 caudal centra, unlike the following ones, lack haemal arches (Fig. 5D). The last ossified caudal ribs likely articulate with the sixth caudal centrum. In BES SC 999, PIMUZ T 4376, and PIMUZ T 1895, caudal centra between the 6th position and the wedge-shaped centra still show small and rounded diapophyses, which are visible up until the tailbend (PIMUZ T 1895), indicating they likely articulated with non-ossified elements (Fig. 5A, D). In the proximal-most caudal centra, the diapophysis contacts the anterior margin of the centrum (at least until the 10th caudal position in BES SC 999), although it does not merge with the cranial articular facet. From the proximal to the caudal-most caudal where a diapophysis is visible, they gradually decrease in size until they are a point-like element in caudal centrum 55 (PIMUZ T 1895). A similar condition is seen in *Shonisaurus popularis* (Camp, 1980) and *Cymbospondylus petrinus* (Merriam, 1908).

The presence of articular surfaces for ribs, as well as the change in the direction of the dorsoventral axis of the neural spines, was used to infer the location of the tailbend in BES SC 999: the positions of the apical centra are inferred as positions 56–60 in the caudal series (Fig. 4F).

*Neural arches and spines*—In PIMUZ T 4376, most of the presacral neural arches are exposed in right lateral view and preserved in articulation (Fig. 4A, B). In PIMUZ T 4376, the element previously described as possible opisthotic (Bindellini et al., 2021) is here reidentified as the atlantal neural arch (Fig. 4A). PIMUZ T 1895 shows a well-preserved articulated series of neural arches in the cranial most portion of the trunk (Fig. 5B). In BES SC 999, the neural arches are also well preserved, although they are sometimes partly covered by other bones (Fig. 4C–G).

Among the studied specimens, only PIMUZ T 4376 shows a well-preserved axis (Fig. 4A); its neural arch has a craniocaudally expanded neural spine, around two times broader than the subsequent spines.

In general, the neural arches possess a triradiate outline in cranial/caudal view. The pedicle is as long anteroposteriorly as the length of the centrum; the medial margin of the articular surface is straight, whereas the lateral margin shows a lateral bulge. This expansion is clearly visible up until the 38th/39th centrum, which are the last two centra where the facet for the neural arch contacts the diapophysis (see description of dorsal centra; Fig. 4D). Prezygapophyses and postzygapophyses are of similar size in the cervical and anterior dorsal vertebrae; in the posterior dorsal and anterior caudal vertebrae, the prezygapophyses project cranially more than the postzygapophyses do caudally (Figs. 4 and 5). The zygapophyses are paired, similar to the condition in *Shastasaurus osmonti* and *Cymbospondylus* (Merriam, 1908; Sander, 1989), but unlike in post-Triassic ichthyosaurs, in which the anterior neural arches show unpaired zygapophyses (e.g., McGowan & Motani, 2003; Moon & Kirton, 2016). The articular surfaces of the zygapophyses are almost horizontal (Figs. 4 and 5).

In lateral view, the dorsal neural spines of *Besanosaurus leptorhynchus* are craniocaudally expanded dorsally, with a generally convex dorsal margin, more rounded than what is visible in *Cymbospondylus buchseri* (Sander, 1989) and *Phantomosaurus neubigi* (Sander, 1997) (this feature is more evident in the caudalmost dorsal vertebrae). The neural spines are inclined at an angle that varies along the vertebral column: cervical and dorsal neural spines are posteriorly inclined at an angle of about 25° from the vertical axis (as also described for *Cymbospondylus buchseri*; Sander, 1989) and near the inferred position of the sacrum the neural spines become more vertical. More caudally, the anterior caudal neural spines are posteriorly inclined at an angle of about 15° (Fig. 5D). The caudal neural spines, in the first half of the tail, are inclined posteriorly almost at an angle of 45°, but posterior to the tailbend, the spines become abruptly shorter and vertically oriented, whereas more caudally their inclination becomes inverted, i.e., the dorsoventral axis of the neural spines changes direction pointing anteriorly (Dal Sasso & Pinna, 1996: fig. 14). Taller neural spines, visible just before the wedge-shaped flexural centra, likely supported a low dorsal lobe of the tail fluke.

*Haemal arches (chevrons)*—In BES SC 999, chevrons are visible starting from the 5th or 6th caudal centrum. In cranial/caudal view most of the chevrons are Y-shaped, but the caudalmost chevrons are V-shaped (Fig. 5A, C, D). In lateral view, the chevrons are straight and the cranialmost ones are dorsoventrally taller than the corresponding centrum (Fig. 5D). Approaching the tailbend, the dorsoventral height of the chevrons gradually decreases, whereas their mediolateral width remains constant (Figs. 4 and 5). Caudally to the tailbend, haemal arches keep decreasing in size, also showing a clear mediolateral width reduction (Fig. 4F, G) if compared to the more anterior chevrons. In both BES SC 999 and PIMUZ T 4376, tiny haemal arches are visible extending almost until the very tip of the tail (Fig. 4G). This condition contrasts with parvipelvians (excluding *Ichthyosaurus communis*) and basal euichthyosaurians (e.g., *Californosaurus perrini*), in which the haemal arches are reduced in size and disappear more cranially (e.g., McGowan & Motani, 2003; Merriam, 1902; Moon & Kirton, 2016).

#### Ribs

In BES SC 999 and PIMUZ T 4376, the ribcage is complete and most ribs are spaced as in vivo; in PIMUZ T 4847 and PIMUZ T 1895 the ribcage is only partly complete (Figs. 2 and 3). At the level of the 55th presacral vertebra, the ribs gradually become shorter and, as a result, the trunk tapers approaching the pelvic region. Post-scapular rib heads are exclusively holocephalous (Figs. 4 and 5), a condition similar to that in *Cymbospondylus* (e.g. Sander, 1989) and other non-euichthyosaurian ichthyosaurs (e.g., Camp, 1980; Merriam, 1902; Nicholls & Manabe, 2004; Shang & Li, 2009). In BES SC 999, the longest rib (articulating with the 29th presacral vertebra) is 46 cm long. In cross-section, the ribs are approximately eight-shaped with a clear furrow running both on the cranial and caudal surface throughout their whole length. The presence of these furrows may be partially the result of trabecular bone collapse, nonetheless they are also clearly visible in PIMUZ T 4376, in which the anatomy of the bones is preserved more three-dimensionally.

*Cervical ribs*—Cervical ribs are dichocephalous (Fig. 4A, C). They are located cranially to the scapulae. The capitulum and tuberculum are round and small, and separated by a small notch. The shafts of the cervical ribs are slightly curved laterally and the distal ends taper into pointed tips. The anterior and posterior furrows of the cervical ribs extend to the level of at least the 8th centrum and are shallower than those in the dorsal ribs, resulting in a less constricted cross-section of the midshaft. In PIMUZ T 4376, the rib articulating with the axis is the shortest among the preserved presacral ribs, being as long as the width of the adjacent centrum. Cervical ribs gradually increase in length, so that the last cervical rib reaches one-third of the length of the longest dorsal rib.

*Dorsal ribs*—Dorsal ribs are holocephalous (Figs. 4 and 5) and located posteriorly to the scapula. They reach their greatest length posterior to the 15th dorsal centrum and maintain a constant length almost up to the sacral region. The head region is slightly sigmoidal in craniocaudal view, similar to what can be observed in *Cymbospondylus buchseri* (PIMUZ T 4351, MSNM V927). The proximal end is also dorsomedially expanded and the articular surface of the head matches the profile of the long and curved (cranially concave) diapophysis. Proximal and distal ends are equally broadened.

*Sacral ribs*—We distinguish two unambiguous pairs of sacral ribs in BES SC 999 (Fig. 4E). These sacral ribs are preserved lying close to each other and are located in close proximity to the ilia. They clearly show an expanded distal end that was likely connected with or located immediately adjacent to the distal portion of the ilium, as in *Guizhouichthyosaurus tangae* (Shang & Li, 2009). The proximal ends of the sacral ribs are also expanded, but not as much as the distal ends. In BES SC 999, the distal articular surfaces of the sacral ribs show a degree of rugosity greater than in the adjacent ribs. Both in the holotype and in PIMUZ T 4376, two additional pairs of ribs with enlarged distal ends are present, but their intermediate morphology (see below) does not allow for their unambiguous identification as sacral ribs. In PIMUZ T 4376, these two further pairs of potential sacral ribs are very short, straight, and possess a thickened midshaft in comparison with the caudalmost dorsal ribs and the rostralmost caudal ribs.

*Caudal ribs*—As mentioned above, the last ossified caudal rib likely articulated with the sixth caudal centrum, as is visible in BES SC 999 (Fig. 4E). Between the 6th caudal centrum and the caudal peak, small and rounded articular surfaces for ribs are visible on the lateral sides of the centra in BES SC 999, PIMUZ T 4376, and PIMUZ T 1895. However, the corresponding caudal ribs are not preserved in any of the three specimens, suggesting that these ribs were likely unossified. The ossified caudal ribs are short, straight, and rod-like, with dorsoventrally expanded proximal and distal ends. The last preserved caudal rib terminates in a pointed distal tip and shows an approximately compressed-conical morphology (Fig. 5D).

#### Gastralia

All specimens preserve gastralia, usually scattered and often broken, but PIMUZ T 1895 and BES SC 999 preserve some articulated elements (Figs. 2 and 3; Fig. S1). Given their state of preservation, it is difficult to estimate the exact number of gastral ribs. Each gastral rib consisted of five elements: one median unpaired element and two lateral paired elements, like in *Cymbospondylus* (Sander, 1989) and *Mixosaurus* (Renesto et al., 2020). The median element is boomerang-shaped with an angle of 135°–140° (145° in *Cymbospondylus buchseri*; Sander, 1989). It possesses a short median anterior process, which is less well-developed in the smaller specimens (PIMUZ T 4376 and PIMUZ T 1895) (Figs. 2 and 3). The element that connects the median to the lateralmost gastralium is a thin recurved bone with pointed medial and lateral ends. The lateralmost element shows a slightly sigmoidal lateral end and a recurved medial end. As reported for the ribs, two shallow furrows are visible on the cranial and caudal surfaces of each gastral element. As in the case of the ribs, these furrows may be to some degree the result of trabecular bone collapse. The gastral basket extended from immediately posterior to the coracoids to the caudalmost region of the trunk.

### Appendicular skeleton

#### Pectoral girdle

*Scapula*—The scapulae are well preserved in BES SC 999 and PIMUZ T 4376 (Fig. 6A–C). In the holotype, the right scapula is exposed in mediodorsal view, whereas the left one is exposed in lateroventral view; in PIMUZ T 4376, we observe the opposite condition. In PIMUZ T 1895 and PIMUZ T 4847, both scapulae are fragmentary and not well preserved (Figs. 3 and 6D).

Overall, the scapula is flat with a relatively thicker, dorsoventrally expanded proximal end. The anterior flange of the scapula is absent, whereas the posterior extension is large and prominent, resulting in the general profile of the scapula resembling an asymmetrical fan. The longitudinal axis of the scapula is oriented at ~ 20° with respect to the glenoid facet. At the proximal end, in medioventral view, the facet for the coracoid extends craniocaudally and measures more than twice the length of the glenoid facet; on the other hand, the glenoid facet has a larger contribution in lateroventral view. The articular facet for the coracoid in the right scapula of BES SC 999 shows a rugose surface indicating the presence of a considerable amount of cartilage in vivo. A small neurovascular foramen is visible perforating the scapular blade anteroproximally. On the visible surfaces, radial striations extend from the ossification centre to the margins of the bone.

The scapula of *Besanosaurus leptorhynchus* closely resembles that of *Guizhouichthyosaurus tangae* (Shang & Li, 2009), although the latter is proportionally slightly shorter proximodistally. However, unlike in *Shastasaurus*, the scapula of *Besanosaurus leptorhynchus* does not possess an anteroproximal notch (Merriam, 1902: pl. 10 and pl. 12) and unlike in ‘*Callawayia*’ *wolonggangense*, the scapula of *Besanosaurus leptorhynchus* does not possess a small anteroproximal extension (Chen et al., 2007: fig. 3B). The scapula of *Besanosaurus leptorhynchus* also differs markedly from the scapulae of *Shonisaurus popularis* (Camp, 1980: fig. 40), *Shonisaurus sikanniensis* (Nicholls & Manabe, 2004: fig. 12), *Callawayia neoscapularis* (McGowan, 1994: fig. 6) and parvipelvian ichthyosaurs, in which the posterior extension of the scapula is reduced and forms an elongate blade (Maisch & Matzke, 2000: char. 71).

*Coracoid*—BES SC 999 preserves a complete left coracoid exposed in ventral view; in PIMUZ T 4376 both coracoids are complete, but only the right one is clearly visible and exposed in dorsal view. PIMUZ T 4847 preserves both coracoids, although they are not well preserved (Fig. 6B).

The coracoids of *Besanosaurus leptorhynchus* are plate-like elements that are craniocaudally longer than mediolaterally broad and possess a deep, cranial notch. As a result, they superficially resemble a broad axe head in outline. The proximal end is more prominent and broader in BES SC 999 and PIMUZ T 4847 than in PIMUZ T 1895 and PIMUZ T 4376. Proximally, the scapular facet and the glenoid facet are in continuity with each other, although the glenoid contribution is almost two times dorsoventrally taller than the scapular facet. The cranial margin of the coracoid is characterised by the presence of an anterior notch that is much deeper in PIMUZ T 4847 than in PIMUZ T 4376, with an intermediate condition visible in BES SC 999. The caudal margin is slightly concave, with a clear posterior notch being absent. The medial margin, which forms the intercoracoid facet (with a possible craniomedial contact for the interclavicle) is long and rounded.

CT images of the left coracoid of BES SC 999 reveal the presence of a slightly concave dorsal surface and a convex ventral surface. As in the scapula, radial striations extend from the ossification centre to the margins of the bone.

As in other shastasaur-grade ichthyosaurs (e.g., *Guizhouichthyosaurus tangae*, Shang & Li, 2009; *Shastasaurus osmonti*, Merriam, 1902; *Shonisaurus* sp., Camp, 1980; Callaway & Massare, 1989; Nicholls & Manabe, 2004), both the cranial and caudal margins of the coracoid are concave in *Besanosaurus leptorhynchus*. However, the coracoid of *Besanosaurus leptorhynchus* is proportionally shorter mediolaterally than in the abovementioned taxa and its distal margin is concave, not straight. The craniocaudal length of the coracoid peduncle (sensu Maisch & Matzke, 2000) is more than half the length of the coracoid itself, similar to *Shastasaurus osmonti* (Merriam, 1908), *Shonisaurus popularis* (Camp, 1980), and *Guizhouichthyosaurus tangae* (Shang & Li, 2009). The cranial (preglenoidal, sensu Maisch & Matzke, 2000) extension of the coracoid in *Besanosaurus leptorhynchus* is greater than the caudal (postglenoidal) portion, as described for other shastasaur-grade ichthyosaurs (e.g., Maisch & Matzke, 2000); however, this latter portion in *Besanosaurus leptorhynchus* is proportionally smaller than in ‘*Callawayia’ wolonggangense* and *Guizhouichthyosaurus tangae* (Chen et al., 2007; Shang & Li, 2009).

*Clavicle—*Both clavicles are well preserved in BES SC 999 and PIMUZ T 4376 (Figs. 2 and 6; Fig. S2). In the latter, these bones are both exposed in dorsal view, whereas in the holotype the right clavicle is exposed in ventral view and the left one is rotated by 180° and exposed in dorsal view.

The clavicles are long and slender, gently broadened at midshaft, and gently curved towards the glenoid region. Proximal ends are slightly expanded whereas distally the clavicles taper to a pointed end. The dorsal surfaces of the clavicles are convex, the ventral ones are concave: in the distal half, this concavity consists of a deep groove that is shallower proximally. As reported in *Guizhouichthyosaurus tangae* (Shang & Li, 2009), the medial margins articulate with the central interclavicle, so that the three elements form a continuous recurved cranial margin. The caudal tip of the clavicle contacts the scapula lateroventrally, as indicated by a specific dorsomedial articulation surface for the clavicle on the scapula. The clavicles of *Besanosaurus leptorhynchus* are similar to those of *Shastasaurus osmonti* (Merriam, 1902), *Guizhouichthyosaurus tangae* (Shang & Li, 2009), and *Shonisaurus popularis* (Camp, 1980), although in the latter taxon they are proportionally much shorter.

*Interclavicle—*The only specimen in which an interclavicle is visible is PIMUZ T 4376 (Fig. 6B; Fig. S3). This bone is T-shaped, with proportionally long lateral processes contacting the clavicles cranially, and what seems to be a broad, proportionally short, triangular caudal process. An ossified rod of the caudal process of the interclavicle is apparently missing. This could be due to the early ontogenetic stage of the specimen, due to its loss during fossilisation, or due to its actual absence. It is unclear if the interclavicle prevented the two clavicles from contacting each other or not. The interclavicle of *Besanosaurus* is similar to that of *‘Callawayia’ wolonggangense* (Chen et al., 2007: fig. 3B). The preserved portion of the interclavicle is also similar to *Guizhouichthyosaurus tangae* (Shang & Li, 2009), except for the absence of the posterior rod. In comparison, the interclavicle of *Mixosaurus cornalianus* (BES SC 1000) has a short and robust caudal process that does not produce a posterior rod (Renesto et al., 2020). Also, the interclavicles of *Besanosaurus leptorhynchus* and *Guizhouichthyosaurus tangae* clearly differ from that of *Shonisaurus sikanniensis*, which possesses a mediolaterally expanded posterior rod and shortened lateral processes (Nicholls & Manabe, 2004).

#### Forefin

None of the specimens show fully articulated and complete forefins; however, the left stylopodium and zeugopodium of BES SC 999 are preserved in articulation. Each element of the forelimb is suboval to rounded in outline, except for the elements that possess an anterior or posterior notch. The zeugopodium of *Besanosaurus leptorhynchus* is ~ 66% the length of the stylopodium. The manus possesses four primary digits (II–V; Motani, 1999) and one accessory digit. The phalanges are well-separated, likely surrounded in vivo by a considerable amount of cartilage, in contrast to the hindlimbs, where the phalanges are tightly packed together and retain rudimentary shafts. The roundness of the forelimb elements is shared both by small and large (PIMUZ T 4376, BES SC 999, PIMUZ T 4847) individuals; this character, which we suspect to be paedomorphic, is also shared with *Guizhouichthyosaurus tangae* (Shang & Li, 2009, 2013), *Guanlingsaurus liangae* (Ji et al., 2013) and to a lesser extent, at least in the phalanges, in other shastasaur-grade ichthyosaurs (Camp, 1980; Chen et al., 2007; Merriam, 1902, 1908).

*Humerus*—PIMUZ T 4376 is the only specimen that preserves both humeri completely. In this specimen, the humeri are slightly craniocaudally broader than proximodistally long (Fig. 6B). The same is true for the left humerus of BES SC 999, but not for the right one, which shows an opposite condition (Table 1). This difference is likely attributable to different stresses that acted on both specimens during the process of fossilisation, resulting in shape distortion caused by compression. PIMUZ T 1895 and PIMUZ T 4847 possess incomplete and fragmentary humeri.

**Table 1:** Humeral proportions of PIMUZ T 4376 and BES SC 999

Specimen number	Craniocaudal/proximodistal length ratio	
	Right humerus	Left humerus
PIMUZ T 4376	1.04	1.05
BES SC 999	0.98	1.09

In dorsal/ventral view, the distal width of the humerus is slightly greater than its proximal width. The anterior flange of the humerus is present but reduced (Motani, 1999). The anterior flange bears a shallow notch in its middle in PIMUZ T 4376, and a proportionally deeper notch is present in BES SC 999. On the dorsal side of the humerus, the head is directed proximodorsally, as in other shastasaur-grade ichthyosaurs (Motani, 1999). On the ventral side, the deltopectoral crest appears to be only slightly raised, but this might be the result of taphonomical compression. Likely for the same reason, the posterodistal tuberosity seems to be flat. A deltopectoral ridge runs distally from the deltopectoral crest towards the centre of the ventral surface.

In BES SC 999, on the dorsal side of the proximal end of the humerus, the facet for the scapula is visible. Symmetrically, on the ventral side, a small portion of the facet for the coracoid is visible. However, both these articular surfaces are not well preserved in the holotype, and in the other specimens these features are not clearly visible. The distal end of the humerus hosts a relatively large, slightly concave, and anterodistally directed facet for the radius; the facet for the ulna is directed posterodistally. Judging from the CT images of BES SC 999, the radial facet is ~ 1.5 times longer than the ulnar facet.

The humerus proportions in *Besanosaurus leptorhynchus* resemble those in *Pessosaurus polaris* (PMU 24585 = PMU R176; Motani, 1999: fig. 3; GB, pers. obs.) and *Guanlingsaurus liangae* (Ji et al., 2013; Sander et al., 2011). In contrast, in *Pessopteryx nisseri* (Wiman, 1910) and *Guizhouichthyosaurus tangae* (Shang & Li, 2009) the humerus is proximodistally longer than craniocaudally wide*.* On the other hand, the humerus of *Besanosaurus leptorhynchus* is more rounded than in *Callawayia neoscapularis* (McGowan, 1994), *Shonisaurus popularis* (Camp, 1980), *Shastasaurus osmonti* (Merriam, 1902, 1908) and ‘*Callawayia*’ *wolonggangense* (Chen et al., 2007), in which the posterior margin of the humerus is proximodistally taller than the anterior margin and distinctly concave.

*Radius*—The radius is the second largest bone in the forefin. Like in the case of the humeri, both radii are complete and well-preserved in BES SC 999 and PIMUZ T 4376 (Fig. 6A–C). The ?right radius is also preserved in PIMUZ T 4847 (Fig. 6D).

The radius lies cranially to the ulna and contacts the humerus proximally: the facet for the latter is straight and long and corresponds to the anterodistally directed radial facet at the distal end of the humerus. The radii in BES SC 999 and PIMUZ T 4376 possess an approximately squared profile in dorsal/ventral view and are only slightly craniocaudally broader than proximodistally long. On the other hand, the ?right radius of PIMUZ T 4847 shows an opposite condition (Table 2). The different proportions of the radii in PIMUZ T 4847 and PIMUZ T 4376 are likely related to ontogeny, taphonomy, or a combination of both. The anterior margin of the radius is characterised by the presence of a proximodistally wide notch in the adults; this notch is shorter and shallower in the juvenile. The caudal margin of the radius is characterised by the presence of a shallow concavity adjacent to the proximal end, which might have accommodated a small portion of the rounded proximocranial margin of the ulna.

**Table 2:** Radial proportions in PIMUZ T 4376 and BES SC 999

Specimen number	Craniocaudal/proximodistal length ratio	
	Right radius	Left radius
PIMUZ T 4376	1.12	1.11
BES SC 999	1.03	1.04
PIMUZ T 4847	0.98	–

The radius of *Besanosaurus leptorhynchus* is most similar to those of *Guizhouichthyosaurus tangae* (Shang & Li, 2013) and *Shastasaurus osmonti* (Merriam, 1902, 1908), which are also subrectangular in outline (although slightly broader craniocaudally than tall proximodistally) and also bear a notch anteriorly. In *Pessosaurus polaris*, *Shonisaurus popularis* and *Callawayia neoscapularis*, the radius is craniocaudally markedly broader than it is proximodistally tall and possesses a cranial margin that is proximodistally longer than the caudal margin, a straight, weakly concave or notched cranial margin, and a weakly concave or notched caudal margin (Camp, 1980; Motani, 1999; Nicholls & Manabe, 2001). In contrast, the radius of *Guanlingsaurus liangae* is suboval in outline, but with the anterior margin also proximodistally longer than the caudal one (Sander et al., 2011).

*Ulna*—Both ulnae are preserved in PIMUZ T 4376 and BES SC 999 (Fig. 6A–C). In the latter specimen, the ulnae are preserved close to their in vivo position, although the left one is incomplete, whereas in PIMUZ T 4376 they are disarticulated. An incomplete ulna is also preserved in PIMUZ T 4847. The ulna is always subcircular in outline and slightly smaller than the radius.

In contrast to *Besanosaurus leptorhynchus*, the ulna in *Guanlingsaurus liangae* is suboval in outline, being anteroposteriorly broader than proximodistally long (Sander et al., 2011). In *Shastasaurus osmonti* and *Callawayia neoscapularis* the ulna is also anteroposteriorly longer than proximodistally broad, but is subrectangular in outline (Merriam, 1902, 1908; Nicholls & Manabe, 2001). Yet another type of ulnar morphology is present in *Guizhouichthyosaurus tangae* and *Shonisaurus popularis*, in which the ulna is proximodistally longer than anteroposteriorly broad (Camp, 1980; Shang & Li, 2009, 2013). In the ulnae of both PIMUZ T 4376 and BES SC 999, the roundness (ratio between the inscribed and the circumscribed circles) equals ~ 0.8. In comparison, this ratio equals 0.67 in *Guizhouichthyosaurus tangae* (Shang & Li, 2009) and 0.61 in *Callawayia neoscapularis* (McGowan, 1994).

**Fig. 7:**
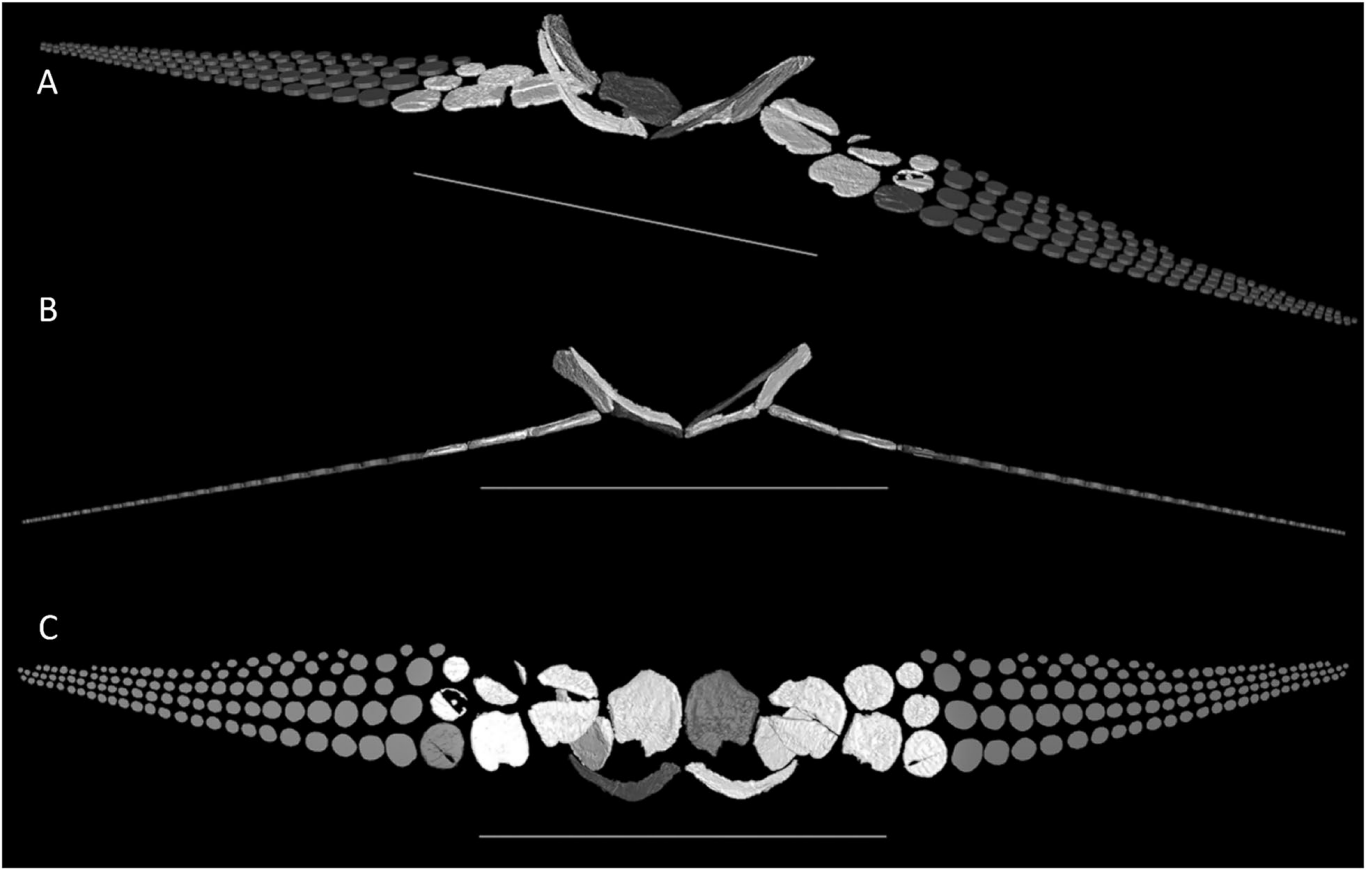
3D reconstruction of the scapular girdle and forefins of *Besanosaurus leptorhynchus* based on the CT scans of BES SC 999 in laterofrontal (**A**), frontal (**B**), and ventral (**C**) view. Original elements in white; elements reconstructed on the basis the available material in grey. Scale bars represent 50 cm. Note that the forefin elements were subject to extreme dorsoventral compression post-mortem and appear much more flattened in the reconstruction than they would be in vivo

*Carpus*—The proximal and distal carpals of *Besanosaurus leptorhynchus* possess a generally subcircular profile and shafts of the bones are not retained. The radiale is the largest among the carpals, with the intermedium being slightly smaller. The ulnare and all other carpal elements are smaller than the intermedium (Figs. 2, 6, and 7).

In BES SC 999, where the carpal elements are preserved relatively close to their original position, three proximal carpals (radiale, intermedium, and ulnare) and four distal carpals can be identified. One small and rounded distal element, likely representing a pisiform, is also visible. It likely articulated caudally with the two carpal rows, similar to the condition in *Guizhouichthyosaurus tangae* (Shang & Li, 2009). The radiale and ulnare are almost perfectly circular, whereas the intermedium seems to be slightly proximodistally longer than craniocaudally wide.

In BES SC 999 and PIMUZ T 4376, the radiale does not bear a notch; only the ?right radiale of PIMUZ T 4847 has a notch on its anterior margin. In BES SC 999, the ?posterior margin of the right intermedium is notched, whereas the left one is rounded like the other carpals. Therefore, the presence of a notch on the cranial or caudal margin of the proximal carpals was variable in *Besanosaurus leptorhynchus*. Among shastasaur-grade ichthyosaurs, a non-notched radiale was reported in *Guizhouichthyosaurus tangae* (Shang & Li, 2009) and *Shastasaurus osmonti* (Merriam, 1902), whereas in *Shonisaurus popularis* both notched and non-notched radialia were reported (Camp, 1980; Sander, 2000).

*Metacarpus and manual phalanges*—Metacarpals and manual phalanges are preserved in BES SC 999, PIMUZ T 4376, and PIMUZ T 4847 (Figs. 2, 3). In BES SC 999, almost all phalanges are preserved and scattered on the surface of the slabs comprising the right forefin. The metacarpals and the manual phalanges are all subcircular in outline. *Besanosaurus leptorhynchus* almost certainly had four primary digits (representing primary digits II–V; Motani, 1999), with digit V possessing markedly smaller phalanges compared to the other three digits. Following this interpretation, *Besanosaurus leptorhynchus* is the stratigraphically oldest ichthyosaur reported to date, showing the loss of primary digit I (Motani, 1999). We estimate the presence of at least 20 phalanges in the longest primary digit (III). The autopodium (estimated to measure ~ 55 cm in BES SC 999) is longer than the stylopodium+zeugopodium. Phalanges are not packed close to each other, but are well separated, likely surrounded in vivo by a considerable amount of cartilage. According to our reconstruction, a few tiny elements belonging to a postaxial accessory digit are also present; these phalanges and their arrangement are similar to those in *Guizhouichthyosaurus tangae*, although in the latter the postaxial accessory digit has a higher number of phalanges (Shang & Li, 2013). Rounded phalanges are also present in *Shastasaurus osmonti*, *Californosaurus perrini* (Merriam, 1902) and *Shonisaurus popularis* (Camp, 1980). However, the latter possesses a notch on the cranial margin of each element of the autopodium, a character absent in *Besanosaurus leptorhynchus.*

#### Pelvic girdle

The ilium of *Besanosaurus leptorhynchus* is similar in size to the other pelvic bones, as in other shastasaur-grade ichthyosaurs (Camp, 1980; Ji et al., 2013; Merriam, 1908; Shang & Li, 2009) and *Callawayia neoscapularis* (Nicholls & Manabe, 2001), but differs from *Cymbospondylus* (Merriam, 1908), *Mixosaurus* (Repossi, 1902) and *Californosaurus perrini* (Merriam, 1902), in which the ilium is markedly smaller than the other pelvic bones.

The pubis and ischium of *Besanosaurus leptorhynchus* are nearly equal in size, with the ischium being slightly smaller than the pubis, as is also the case in *Cymbospondylus* (Merriam, 1908), *Guizhouichthyosaurus tangae* (Shang & Li, 2009), and *Shonisaurus popularis* (Camp, 1980), but differs from the condition in *Shastasaurus osmonti* (Merriam, 1902), *Guanlingsaurus liangae* (Ji et al., 2013) and *Callawayia neoscapularis* (Nicholls & Manabe, 2001), in which the two bones are sub-equal in size, and *Californosaurus perrini* (Merriam, 1902), in which the ischium is slightly larger than the pubis.

**Fig. 8:**
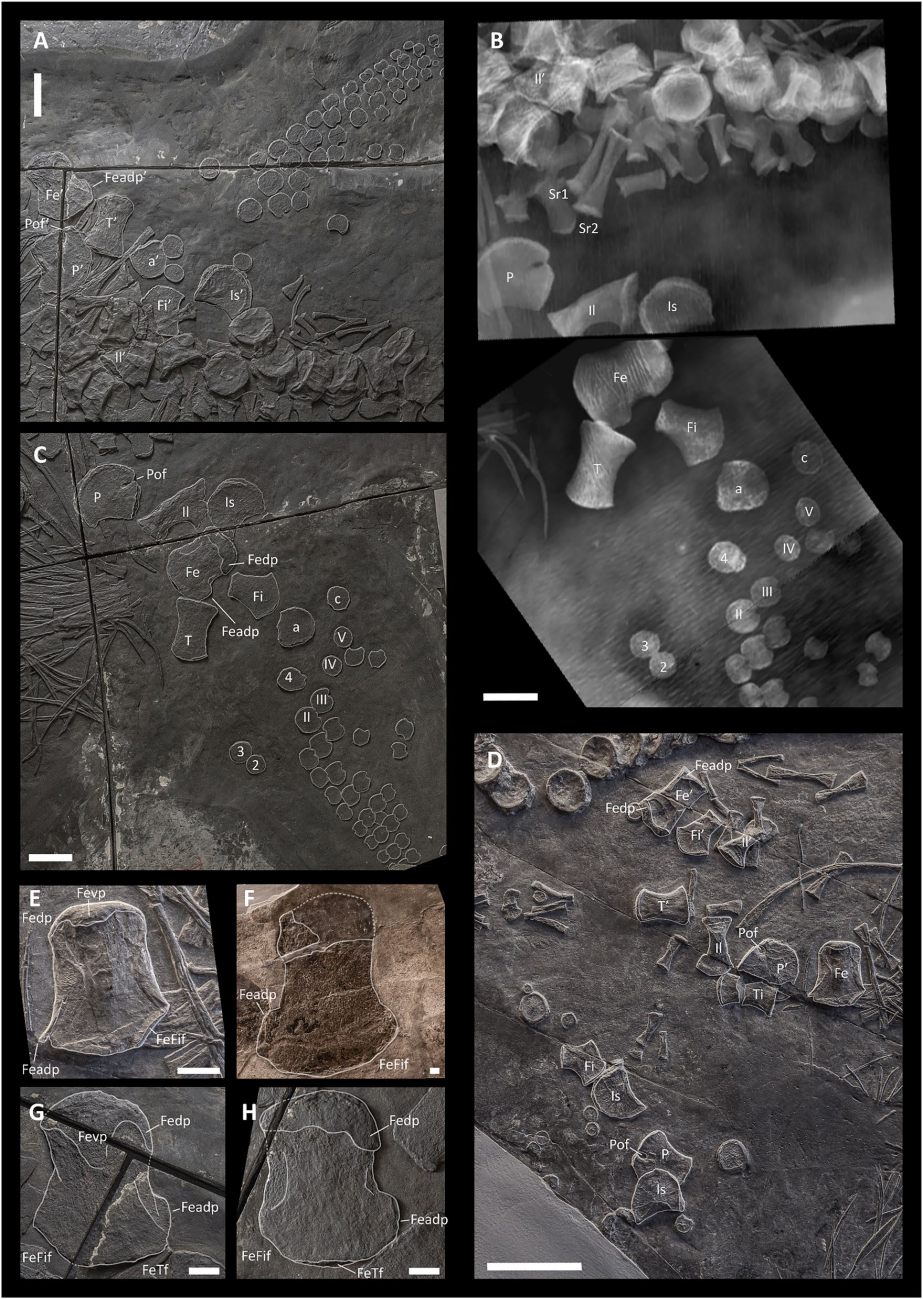
Left (**A**) and right (**C**) hindfin and pelvic girdle elements of BES SC 999; (**B**) CT scan rendering of the right pelvic girdle and forefin elements of BES SC 999; (D) hindfin and pelvic girdle elements of PIMUZ T 4376; (**E**) right femur of PIMUZ T 4376; (**F**) femur of PIMUZ T 4847; (**G**, **H**) left and right femora of BES SC 999. *a* astragalus, *c* calcaneum, *Fe* femur, *Feadp* femur anterodistal process, *Fedp* dorsal process of the proximal epiphysis of the femur, *FeFif* femoral facet for the fibula, *FeTf* femoral facet for the tibia, *Fevp* ventral process of the proximal epiphysis of the femur, *Fi* fibula, *Il* ilium, *Is* ischium, *P* pubis, *Pof* obturator foramen of the pubis, *Sr* sacral rib, *T* tibia; 2, 3, and 4, distal tarsals; II, III, IV, and V, metatarsals. The apostrophe (‘) always indicates a left element. Scale bars represent 5 cm in **A**–**D**, and 1 cm in **E**–**H**

*Ilium*—The ilia are well preserved in BES SC 999 and PIMUZ T 4376. In PIMUZ T 4847, only an incomplete element resembling the ?left ilium is preserved (Fig. 8A, B, and D). In BES SC 999, the left ilium is obscured from external view by other bones but is well visible in CT images (Fig. 8).

The ilium has a craniocaudally expanded proximal end that participated in the acetabulum and a craniocaudally expanded distal end that likely contacted the expanded distal ends of the two pairs of sacral ribs, as inferred for *Guizhouichthyosaurus tangae* (Shang & Li, 2009). The distal end of the ilium is craniocaudally wider than the proximal end, similar to *Mixosaurus* (Repossi, 1902), *Shastasaurus osmonti* (Merriam, 1902, 1908), and adult *Guanlingsaurus liangae* (Ji et al., 2013), but is different from the condition in *Cymbospondylus* (Merriam, 1908), *Guizhouichthyosaurus tangae* (Shang & Li, 2009), juvenile *Guanlingsaurus liangae* (Ji et al., 2013), *Callawayia neoscapularis* (Nicholls & Manabe, 2001) and *Californosaurus perrini* (Merriam, 1902), in which the proximal and distal ends of the ilium are of approximately equal width in lateral view. In BES SC 999, both the proximal and distal ends are also highly rugose, suggesting the presence of cartilage in vivo. The shaft of the ilium appears to have been lateromedially flattened relative to the likely thickened proximal end in vivo (compare with Camp, 1980). The right ilium of BES SC 999 and the ?left ilium of PIMUZ T 4847 are preserved in approximately anterolateral/posterolateral view, although they have undergone severe compression. The ilia are slightly curved so that the preserved concave margins correspond to their medial surfaces (compare with Camp, 1980: fig. 51). This is similar to *Shastasaurus* (Merriam, 1908: pl. 16, fig. 4) and recalls the basal ichthyopterygian ilium morphology also present in *Utatsusaurus* (Motani, 1998). A similar curvature of the ilium is also preserved in adult specimens of *Guanlingsaurus* (Ji et al., 2013: fig. 5) and *Callawayia neoscapularis* (Nicholls & Manabe, 2001: fig. 11). The curvature is less pronounced in *Californosaurus perrini* (Merriam, 1902). In PIMUZ T 4376 this curvature is not visible because the ilium is preserved in lateral view; in BES SC 999, the left ilium is preserved in the form of a straight element as well.

*Pubis*—Both pubes are well preserved in BES SC 999 and PIMUZ T 4376 (Fig. 8); a poorly preserved element resembling a pubis is also visible in PIMUZ T 4847.

The pubis is plate-like. Its anterolateral margin is concave, whereas the rest of the bone shows a suboval outline interrupted only by the aperture of the obturator foramen on its caudal margin. The proximal margin, probably bordering one-third of the cranioventral margin of the acetabulum, is dorsoventrally slightly thicker than the rest of the bone. The obturator foramen opens to the caudal margin in all examined specimens. As reported in previous studies (McGowan & Motani, 2003; Motani, 1999; Dal Sasso & Pinna, 1996), the obturator foramen shows an elongated, suboval outline, being almost entirely enclosed within the pubis in the holotype BES SC 999, and slightly open in PIMUZ T 4376. The condition in PIMUZ T 4847, the largest specimen, is uncertain. Among basal Merriamosauria, *Guizhouichthyosaurus tangae* (Shang & Li, 2009) shares the most similar pubis morphology with *Besanosaurus leptorhynchus*. *Shastasaurus* (Merriam, 1902) also shows a similar morphology but possesses a much more rounded medial margin both in the pubis and the ischium. In dorsal and ventral view, the pubis of *Besanosaurus leptorhynchus* recalls the pubis of *Californosaurus perrini* in outline, but the latter shows a much wider aperture of the obturator foramen (Merriam, 1902). A relatively narrow obturator foramen is also present in *Shastasurus osmonti* (Merriam, 1908: fig. 73; Dal Sasso & Pinna, 1996: fig. 21), but it is still broader than that in *Besanosaurus leptorhynchus*.

*Ischium—*Both ischia are well preserved in BES SC 999 and PIMUZ T 4376 (Fig. 8); a possible ischium is also preserved in PIMUZ T 4847. The ischium is a plate-like, fan-shaped bone, which is slightly smaller than the pubis. In ventral and dorsal view, the bone forms a convex anteromedial/ventromedial margin and a concave caudolateral margin. The proximal end of the bone appears dorsoventrally slightly thicker than the rest of it and forms the caudoventral margin of the acetabulum.

Overall, the morphology of the ischium of *Besanosaurus leptorhynchus* is similar to that of basal Merriamosauria, in which this bone is preserved [*Guizhouichthyosaurus tangae* (Shang & Li, 2009); *Shastasaurus pacificus* (Merriam, 1908); *Shonisaurus* *popularis* (Camp, 1980); *Californosarus perrini* (Merriam, 1902)]. In contrast to basal ichthyopterygians (Motani et al., 1998, 2014; Wiman, 1929), some mixosaurids (e.g., Brinkmann, 2004), and *Toretocnemus* (Merriam, 1903), the medial symphysis between the pubes and ischia in *Besanosaurus leptorhynchus* is not straight and well defined, and is similar to the condition in *Cymbospondylus*, other shastasaur-grade ichthyosaurs, *Callawayia neoscapularis,* and *Californosaurus perrini*, which all possess concave ventromedial margins of the pubis and ischium (Merriam, 1902, 1908; Nicholls & Manabe, 2001).

#### Hindfin

Only BES SC 999 and PIMUZ T 4376 preserve both hindfins (Figs. 2, 3). The hindfins are complete in the holotype, whereas most of the autopodia are missing in the latter specimen. In PIMUZ T 4847, only some proximal elements of the hindfins are preserved. Based on the preserved elements and our reconstruction, the hindlimb is about 70% the length of the forelimb. The hindfin possesses four digits representing primary digits II–V (McGowan & Motani, 2003; Shang & Li, 2013). Preaxial and postaxial accessory digits are absent, in contrast to *Guizhouichthyosaurus tangae*, in which a preaxial accessory digit is variably present (Shang & Li, 2013). Moreover, in contrast to the forefin, the phalanges in the hindfin are more tightly packed together and constricted in the middle (retaining a rudimentary shaft), showing a condition more similar to Mixosauridae (e.g., Repossi, 1902; Brinkman, 2004) than to *Guizhouichthyosaurus tangae* (Shang & Li, 2009) and *Shonisaurus popularis* (Camp, 1980), in which the phalanges are suboval to subcircular in outline and lack mid-shaft constrictions.

*Femur*—Femora are preserved in BES SC 999, PIMUZ T 4376, and PIMUZ T 4847, although in the latter specimen they are incomplete (Fig. 8E–H). In BES SC 999 the right femur is preserved in dorsal view, whereas the left femur is preserved in ventral view. However, as a result of extreme compression, the preserved cranial and caudal margins of the femora might not precisely correspond to their actual cranial and caudal margins. In PIMUZ T 4376 the right femur is preserved in ventral view, whereas the left femur is preserved in cranial view. In PIMUZ T 4847 the orientation of the femora is unclear.

In dorsal and ventral view, the femur shows concave cranial and caudal margins; despite this, the midshaft is only slightly constricted. The craniocaudal length of the proximal end is shorter than that of the distal end. On the proximal end, well-developed dorsal and ventral processes are present. The dorsal process is positioned cranioproximally, whereas the ventral process is located more centrally (Maxwell et al., 2012). Both processes extend for approximately one-third of the femoral proximodistal length. Two distal facets for the tibia and fibula, which are in continuity with each other, can be distinguished at the distal end of the bone. The fibular facet is shorter, faces caudodistally, and is inclined at an angle of > 45° with respect to the proximodistal axis of the femur. The facet for the tibia is larger and faces entirely distally. Anterodistally, the femur forms a distinct flange, also seen in *Pessopteryx* (Wiman, 1910).

In dorsal and ventral view, the femoral shaft of *Besanosaurus leptorhynchus* looks craniocaudally broader than the femoral shafts of *Guanlingsaurus liangae* (Sander et al., 2011), *Shonisaurus popularis* (Camp, 1980), and *Californosaurus perrini* (Merriam, 1902). Also, in the three latter taxa, the proximal end is proportionally shorter craniocaudally than in *Besanosaurus leptorhynchus*. Overall, among shastasaurid taxa, the femur of *Besanosaurus leptorhynchus* is most similar to femur type A of *Guizhouichthyosaurus tangae* (Shang & Li, 2013).

*Tibia*—Both tibiae are preserved in BES SC 999, PIMUZ T 4376, and PIMUZ T 4847 (Figs. 2, 3, and 8). In the holotype, the left one is still close to its in vivo position, whereas the right one is markedly dislocated. In PIMUZ T 4376 and PIMUZ T 4847, the tibiae are disarticulated and better preserved in the former.

The proximal end of the tibia is straight in dorsal/ventral view and craniocaudally longer than the convex distal end. The caudalmost portion of the distal end likely contacted the astragalus, as in articulated hindfins of *Guizhouicthhyosaurus tangae* (Shang & Li, 2013). In dorsal and ventral view, the shaft is straight and relatively long, with gently concave cranial and caudal margins. This is in contrast to the condition in *Guizhouichthyosaurus tangae* (Shang & Li, 2013), *Guanlingsaurus liangae* (Sander et al., 2011), *Shastasaurus osmonti* (Merriam, 1902), *Shonisaurus popularis* (Camp, 1980), and *Californosaurus perrini* (Merriam, 1902), in which the tibial shaft is proportionally shorter and thicker, and the anterior margins are deeply concave.

*Fibula*—Both fibulae are preserved in BES SC 999, PIMUZ T 4376, and PIMUZ T 4847 (Figs. 2, 3, and 8). Like the tibia, the left fibula is still close to its in vivo position in the holotype.

The proximal end of the fibula is craniocaudally much smaller than the distal one. The facets for the calcaneum and the astragalus are wide, almost equal in size, and in continuity with each other. The separation between the two facets is not well defined, although they are symmetrically inclined at an angle of ~ 45° with respect to the proximodistal axis of the bone. In vivo, the tibia and fibula were likely completely separated from each other, as none of the two bones show facets for mutual articulation.

Overall, the general shape of the fibula of *Besanosaurus leptorhynchus* is similar to that of *Cymbospondylus* (Merriam, 1908) and most other shastasaur-grade ichthyosaurs (e.g., Merriam, 1902; Sander et al., 2011; Shang & Li, 2013), with the exception of *Shonisaurus popularis*, in which the fibula is proportionally shorter proximodistally and produces a prominent distal flange (Camp, 1980).

**Fig. 9:**
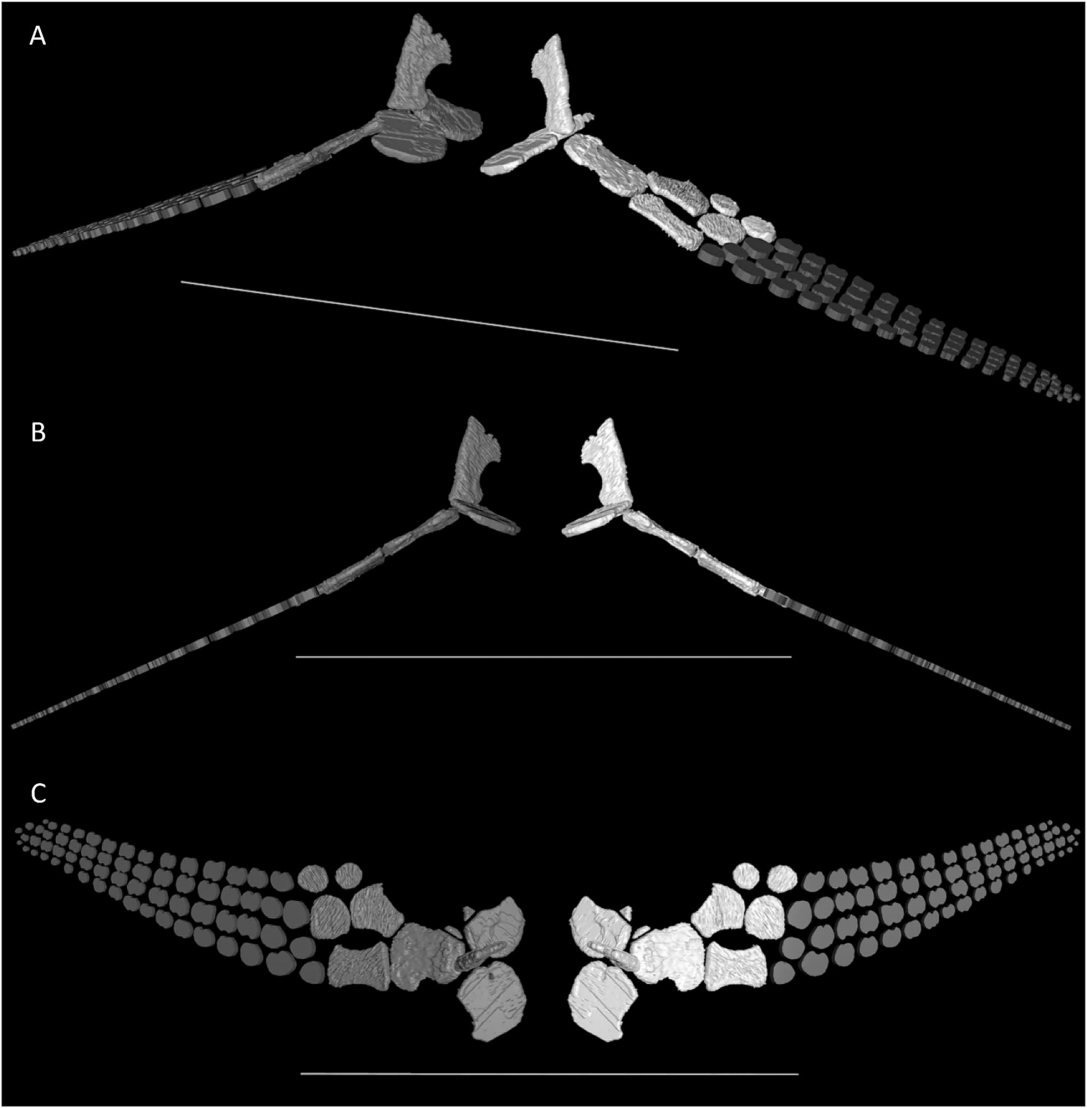
3D reconstruction of the pelvic girdle and hindfins of *Besanosaurus leptorhynchus* based on the CT scans of BES SC 999 in laterofrontal (**A**), frontal (**B**), and dorsal (**C**) view. Shang & Li (2013: fig. 1) and McGowan & Motani (2003: fig. 70) were used as references for hindfin reconstruction. Original elements in white; elements reconstructed on the basis of the available material in grey. Scale bars represent 50 cm. Note that the pelvic girdle and hindfin elements were subject to extreme dorsoventral compression during fossilisation and appear much more flattened in the reconstruction than they would be in vivo

*Tarsus and metatarsus—*BES SC 999 possesses two proximal tarsals: the astragalus and the calcaneum. The presence of ossified tarsals is uncertain in PIMUZ T 4376 due to incomplete preservation of the hindfins. The astragalus is craniocaudally longer than proximodistally tall and much larger than the calcaneum, which is subcircular in outline (Figs. 8, 9). The general shape and proportions of the tarsal bones resemble those of *Guizhouichthyosaurus tangae* (Shang & Li, 2009, 2013) and *Guanlingsaurus liangae* (Ji et al., 2013; Yin et al., 2000), but differ from *Shastasaurus osmonti*, in which the astragalus is also larger than the calcaneum, but both tarsals are subcircular in outline (Merriam, 1908).

In the *Besanosaurus leptorhynchus* holotype, three elements are identified as distal tarsals (distal tarsals 2–4) and four elements are identified as metatarsals (metatarsals II–V). Distal tarsals 2 and 3 are subcircular in outline and much smaller than the suboval distal carpal 3. Metatarsals II and III possess a rounded anterior margin and a shallow notch on the caudal margin. Metatarsals IV and V are subcircular in outline and do not bear any notches. According to our reconstruction, metatarsal V contacted the distal portion of the calcaneum as in several Triassic ichthyosaurs (e.g., McGowan & Motani, 2003). This differs from the condition reported by Shang & Li (2009, 2013) for *Guizhouichthyosaurus tangae*, in which distal tarsal 4 contacts the distal part of the calcaneum and metatarsal V lies in the same row with the other metacarpals.

Pardo-Pérez et al., (2020: fig. 1g, h) reported a case of ankylosis in the hindlimb metapodial elements II and III of BES SC 999. We confirm that a small portion of the caudal margin of metatarsal II is fused to the cranial margin of metatarsal III.

*Pedal phalanges*—In BES SC 999, the pedal phalanges retain rudimentary shafts, showing an anterior and/or a posterior shallow notch (Fig. 8A, C), whereas in PIMUZ T 4376 they are mostly rounded in outline, except for the largest proximal phalanges, which bear anterior notches (Fig. 3). These differences between the two specimens are attributed to their different ontogenetic stages.

In BES SC 999, most of the pedal phalanges are preserved in life position, being in close contact or only slightly separated from each other. This provides evidence that, in *Besanosaurus leptorhynchus*, the pedal phalanges were closely packed and that the relative amount of interphalangeal cartilage was much lower in the hindfin than in the forefin (see above). As a result, the hindfins might have been stiffer than the forefins. This feature contrasts with the condition seen in *Guizhouichthyosaurus* (Shang & Li, 2009), where manual and pedal phalanges show a similar degree of spacing and the pedal phalanges are not preserved in contact with each other. Furthermore, even in large (adult) specimens of *Guizhouichthyosaurus* (Shang & Li, 2009, 2013), all of the pedal phalanges appear to be rounded. Therefore, the pes of adult *Besanosaurus leptorhynchus* appears to be more similar to that of *Mixosaurus*, in which the pedal phalanges retain constricted shafts and are tightly packed (e.g., Brinkmann, 2004; Renesto et al., 2020; Repossi, 1902), than to all other shastasaur-grade ichthyosaurs, in which the pedal phalanges are circular in outline (e.g., Camp, 1980; Shang & Li, 2013; Yin et al., 2000).

## Phylogenetic analysis

We updated the scores of *Besanosaurus leptorhynchus* in the matrix published by Bindellini et al. (2021) based on our revision of the postcranium (Files S1, S2). The analysis was performed in TNT 1.5 (Goloboff & Catalano, 2016) with memory set to hold 99,999 trees; the traditional search option (1000 replications of Wagner trees, TBR branch swapping algorithm with 10 trees saved per replication) was used, followed by an additional round of TBR branch swapping. Bremer support values were calculated by retaining trees suboptimal by 10 steps.

The analysis resulted in 14,480 MPTs of 717 steps (CI 0.361, RI = 0.787). The topology of the majority rule consensus tree is similar to that published by Bindellini et al. (2021), except for a few differences (for strict consensus, see Fig. S4; for 50% majority rule consensus, see Fig. S5). Therefore, the updated scores from the postcranial skeleton of *Besanosaurus leptorhynchus* corroborate the results of some previous phylogenetic analyses, in which “shastasauridae” were recovered as a grade and not a clade (e.g., Maisch & Matzke, 2000; Sander, 2000; Sander et al., 2011; Moon, 2019; Moon & Stubbs, 2020; Sander et al., 2021; contra e.g., Ji et al., 2013, 2016; Jiang et al., 2016; Motani et al., 2017; Huang et al., 2019).

## Discussion

### Affinities of *Besanosaurus* with *Pessopteryx* and *Pessosaurus*

Among Triassic ichthyosaurs, *Besanosaurus leptorhynchus* shows some similarities with fossils attributed to *Pessopteryx* and *Pessosaurus* from the Early–Middle Triassic of Svalbard (Wiman, 1910; Maisch & Matzke, 2000: 85–86; McGowan & Motani, 2003: 127–128, 135–136; Maxwell & Kear, 2013). The humeri referred to *Pessopteryx nisseri* are similar to *Besanosaurus leptorhynchus*, although they do not show a notched anterior margin, and they appear clearly proximodistally longer than craniocaudally wide (e.g., Wiman, 1910: taf. VIII, figs. 1, 2; G.B. pers. obs. 2021). An exception is PMU 24592b (Wiman, 1910: taf. VIII, fig. 3; G.B. pers. obs. 2021), which shows a notched anterior margin and proportions more similar to those of BES SC 999.

The femora of *Besanosaurus leptorhynchus* are also similar to those of *Pessopteryx nisseri*: the anterodistal process occurs in both taxa but seems to be slightly less prominent in femora originally referred to *Pessopteryx* (e.g., PMU 24602, 24603; PMUR 1000–1007; G.B. pers. obs. 2021). The proportions of this process vary in other isolated femora from Svalbard, demonstrating a continuous range (e.g., PMU 24602, 24603; PMUR 1000–1007), and possibly indicating some intraspecific variation. PMU 24585 (Wiman, 1910: taf. VII, fig. 3), a femur attributed to *Pessosaurus polaris*, was described as being more similar to PIMUZ T 4376 than to BES SC 999; this is due to the fact that in both PIMUZ T 4376 and PMU 24585 an anterodistal process was considered to be missing (McGowan & Motani, 2003: 128). However, in both specimens the anterodistal portion of the femur is broken and incomplete (G.B. pers. obs. 2021) and therefore the resemblance between the two specimens must be considered an artefact: in both cases the facet for the tibia is incomplete and the anterodistal process is almost completely missing. Furthermore, the fracture is partly filled by plaster in PIMUZ T 4376 (G.B. pers. obs. 2021).

PMU 24584 (PMUR 176 in Wiman, 1910: taf. VII, fig. 2), a specimen consisting of a coracoid, a radius, a humerus, and a podial element attributed to *Pessosaurus polaris*, was considered similar to PIMUZ T 4376 by Maisch & Matzke (2000), who tentatively referred the specimen to *Mikadocephalus* cf. *gracilirostris*, a junior synonym of *Besanosaurus leptorhynchus* (Bindellini et al., 2021; McGowan & Motani, 2003). The radius of PMU 24584 is proximodistally relatively shorter than those of any other *Besanosaurus leptorhynchus* specimens examined in this study (craniocaudal/proximodistal length ratio equals 0.7 in PMU 24584; in *Besanosaurus leptorhynchus* it ranges from 1.03 to 1.12—Table 2), showing proportions more similar to those of *Shastasaurus osmonti* (Merriam, 1902: pl. 11; ratio equals 0.8). Furthermore, the humerus of PMU 24584 is also relatively shorter proximodistally than that of PIMUZ T 4376 and all other examined specimens of *Besanosaurus leptorhynchus* (craniocaudal/proximodistal length ratio equals 0.9 in PMU 24584; in *Besanosaurus leptorhynchus* it ranges from 0.98 to 1.09—Table 1) and is slightly more similar in proportions to the humerus of *Guanlingsaurus* (Ji et al., 2013: fig. 3; ratio equals 0.82).

Another aspect worth noting is that among the several phalanges attributed to *Pessopteryx* or *Pessosaurus* (e.g., Wiman, 1910: taf. VIII; G.B. pers. obs. 2021) there were no phalanges with retained shafts. The absence of large phalanges with retained shafts in the Svalbard material indicates the likely absence of *Besanosaurus leptorhynchus* from this fauna.

In conclusion, although the presence of *Besanosaurus leptorhynchus* and its possible synonymy with *Pessopteryx* and *Pessosaurus* was previously proposed, in light of the anatomical observations mentioned above, we do not consider *Besanosaurus leptorhynchus* as present in the Triassic Svalbard ichthyosaur fauna and do not consider *Besanosaurus* a possible junior synonym of either of the two taxa (Maisch, 2010; Maisch & Matzke, 2000; McGowan & Motani, 2003). Nonetheless, the similarities between *Besanosaurus leptorhynchus* and material referred to *Pessosaurus* and *Pessopteryx* suggest that large shastasaur-grade ichthyosaurs similar and possibly closely related with *Besanosaurus leptorhynchus* were already present in the Early–Middle Triassic of Svalbard. More complete material is needed to scrutinize the taxonomy and phylogenetic position of *Pessosaurus* and *Pessopteryx*, and to determine whether these two taxa served as ‘waste-basket’ taxa, encompassing isolated material belonging to shastasaur-grade ichthyosaurs, cymbospondylids, mixosaurids, or even omphalosaurids (Qiao et al., 2022).

### Remarks on forefin and hindfin ossification in *Besanosaurus*

Xie et al. (2020) demonstrated that in some odontocete limbs, secondary ossification centres (SOCs) are reduced or absent, contrary to the condition in terrestrial mammals, which results in the autopodial bones becoming widely spaced. The authors suggested that the return of cetaceans to an aquatic environment was associated with a gradual reduction in the size of SOCs in some lineages or even a complete loss in some species. In orcas (Xie et al., 2020: fig. 4), phalanges in the forefin are short and round, completely lack SOCs, and are separated from each other by a large amount of (fibro-)cartilage and connective tissue. Wide spacing of rounded autopodial elements is also seen in the forefins of *Besanosaurus leptorhynchus*, but not in the hindlimbs. As in other ichthyosaurs, these marine reptiles did not have secondary ossification centres, and instead, the change to more unified rounded autopodial bone shapes was achieved by loss of perichondral/periosteal bone in the ‘shaft regions’ of these elements (e.g., Caldwell, 1997). Because of the wide spacing of the individual elements, we hypothesise they were also set in a (fibro-)cartilaginous rich connective tissue, similar to the one seen in *Orcinus orca*. The phalanges gradually became more closely packed throughout the evolution of Ichthyosauria, until they became polygonal and tightly packed in both the fore- and hindfins in Parvipelvia (e.g., McGowan & Motani, 2003).

### Testing the swimming mode of *Besanosaurus*

To investigate the swimming mode of *Besanosaurus leptorhynchus* and sympatric ichthyosaurian taxa, we expanded on the body outline analysis originally published by Motani et al. (1996), as detailed in the Methods section. Anguilliform swimmers show a low H/L ratio and a low fineness ratio and are grouped in the lower left corner of the body outline morphospace, whereas the thunniform swimmers show a high H/L ratio and a high fineness ratio and are clustered in the top right corner (Motani et al., 1996).

The resulting body shape morphospace was divided by two lines (a and b), which define four morphospace regions. The ichthyopterygian taxa, in which a dorsal fin has been reported or inferred, occupy the area of the morphospace above line a. These include parvipelvians and *Mixosaurus*, but also all of the fishes included in the analysis. Basal Ichthyopterygia, Cymbospondylidae, and shastasaur-grade ichthyosaurs occupy the region of the morphospace below line a. Line b divides all those taxa with a caudal fluke (tail height sensu Motani et al., 1996) dorsoventrally taller than their body height (on the right), and taxa that have a tail dorsoventrally shorter than their body height (on the left). Parvipelvians and shastasaur-grade ichthyosaurs occupy the area of the morphospace to the right of line b, whereas more basal ichthyopterygians are plotted on the left.

*Besanosaurus leptorhynchus* falls close to the other shastasaur-grade ichthyosaurs included in this analysis, as well as to *Cymbospondylus*. The fineness ratio of *Besanosaurus leptorhynchus* is similar to the one obtained for *Cymbospondylus*, but the H/L ratio of the caudal fluke is more similar to that of *Gualingsaurus.* Nonetheless, in *Cymbospondylus* this ratio might be affected to some extent by the lack of data regarding the presence and the height of a dorsal lobe of the tail fluke. All non-parvipelvian ichthyosaurs fall inside the 95% confidence ellipse fitted to data for 94 species, belonging to 14 families of sharks (Motani et al., 1996: fig. 2a). Among shastasaur-grade ichthyosaurs included in our analysis, *Gualingsaurus liangae* and *Guizhouichthyosaurus tangae* plot inside the carcharhinid ellipse, whereas *Besanosaurus leptorhynchus* fall just outside of it. The very long tail of *Besanosaurus leptorhynchus* causes it to plot within the scyliorhinid ellipse, close to *Cymbospondylus*. *Utatsusaurus* falls within the scyliorhinid ellipse, and *Chaohusaurus geishanensis* plots just outside of it. However, the original reconstruction of a specimen of *Chaohusaurus chaoxianensis* from Motani et al. (1996) falls inside the scyliorhinid ellipse, i.e., among anguilliform swimmers. Among Triassic ichthyosaurs, solely *Mixosaurus* falls outside the carcharhinid and selachimorph ellipse, close to odontaspid and sphyrenid sharks, which possess the lowest fin H/L ratio among the fishes included in the dataset of Motani et al. (1996).

**Fig. 10:**
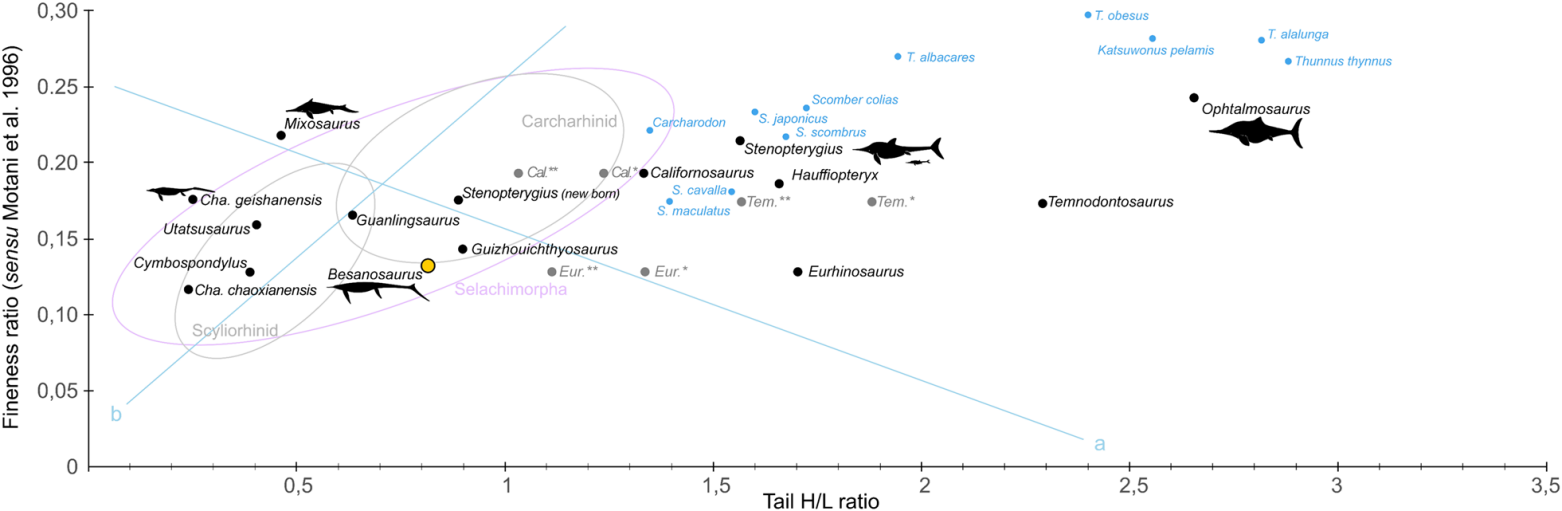
Graph showing the body shape distribution of selected ichthyopterygians and fishes. Tail fin H/L ratio is fluke height divided by fluke length (x-axis), fineness ratio is body height—excluding the dorsal fin—divided by prefluke length (y-axis). Diagram redrawn from Motani et al. (1996) with additional taxa plotted (see text, Fig. S1 and Tab. S1 for details). For *Californosaurus*, *Temnodontosaurus*, and *Eurhinosaurus*, “*” designates a reconstruction in which the dorsal lobe of the caudal fluke is 50% the height of the ventral one, “**” designate the reconstruction in which it is 25% of the height of the ventral lobe

*Stenopterygius* clusters close to Lamniformes (Carcharhinidae), i.e., carangiform swimmers. A new born *Stenopterygius,* clusters closer to more anguilliform swimmers*.* The rest of the parvipelvian ichthyosaurs plot closer to the area occupied by thunniforms, with *Ophthalmosaurus* clustering closer to tuna than to any other ichthyosaur. On the other hand, Triassic ichthyosaurs appear to cluster among subcarangiform and anguilliform swimmers, with *Mixosaurus* being almost an outlier: this taxon possesses the highest fineness ratio among Triassic ichthyosaurs (Fig. 10).

### Remarks on the swimming style of *Besanosaurus*, *Cymbospondylus*, and *Mixosaurus*

**Fig. 11:**
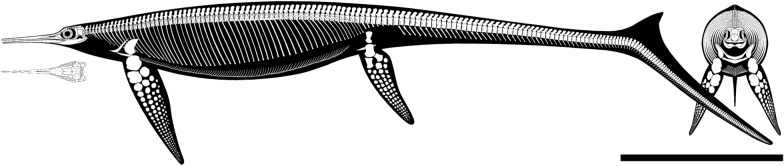
Full skeletal reconstruction of *Besanosaurus leptorhynchus*. Size and proportions are based on the holotype BES SC 999. Scale bar equals 1 m. Line drawing by Marco Auditore

The fineness ratio of *Besanosaurus leptorhynchus* (updated skeletal reconstruction given in Fig. 11; Fig. S7) is more similar to that of *Cymbospondylus*, which clusters among anguilliform swimmers, than to the rest of the shastasaur-grade ichthyosaurs (*Shonisaurus, Guanlingsaurus, Guizhouichthyosaurus*). However, the inferred tail H/L ratio of *Besanosaurus leptorhynchus* is more similar to that of *Guizhouichthyosaurus* than to *Cymbospondylus*. This indicates that *Besanosaurus leptorhynchus* had body proportions intermediate between these taxa, with a trunk shape similar to that of *Cymbospondylus* and a tail more similar to that of shastasaur-grade ichthyosaurs. The inference that *Besanosaurus leptorhynchus* might have swum in a way intermediate between *Cymbospondylus* and more derived ichthyosaurs is supported by the relative phylogenetic position of these taxa (e.g., Bindellini et al., 2021; Ji et al., 2016; Maisch & Matzke, 2000; Moon, 2019; Moon & Stubbs, 2020; Motani et al., 2017; Sander et al., 2011) and by the morphology and position of the rib facets along the vertebral column. Slijper (1936) reported the reduction of the rib count and the replacement of sternal and dichocephalous ribs by floating ribs and holocephalous ribs in the early evolution of whales, associating this trend with an increased thoracic flexibility needed by early whales to swim and dive. Furthermore, in Jurassic ichthyosaurs, ribs are dichocephalous cranially and holocephalous caudally and the transition occurs posterior to the 40th centrum (Buchholtz, 2001), whereas in Triassic ichthyosaurs dichocephalous ribs are present almost exclusively in the cervical region (e.g., Camp, 1980; Fröbisch et al., 2006; Merriam, 1902; Sander, 1989; Shang & Li, 2009). Buchholtz (2001) concluded that dichocephalous ribs have less freedom of movement than holocephalous ones and that the transition point marks the transition from a rigid to a more flexible body region. This hypothesis also supports the idea that Triassic ichthyosaurs were more anguilliform or subcarangiform swimmers, whereas parvipelvians were carangiform and thunniform swimmers.

Among the ichthyosaurs from the Besano Formation, the dimensions and position of the diapophyses in the dorsal vertebrae of *Cymbospondylus buchseri* most closely resemble the caudalmost dorsals and cranialmost caudals of *Besanosaurus leptorhynchus*. These centra are characterised by the presence of small and round diapophyses, that could have granted a greater range of movement to the ribcage. The fact that such morphology appears more cranially in *Cymbospondylus* than in *Besanosaurus leptorhynchus* is consistent with a more anguilliform swimming style in *Cymbospondylus*. In *Mixosaurus*, a second facet for the articulation of dichocephalous ribs reappears on the sides of the centra close to the pelvis (e.g., Brinkman, 2004). This condition, which contrasts with that in *Besanosaurus leptorhynchus* and *Cymbospondylus*, could have granted more stiffness to the dorsum. In support of this hypothesis, we point out that *Mixosaurus*, among Triassic ichthyosaurs, is the only taxon for which a dorsal fin has been reported so far (Renesto et al., 2020). This taxon also shares a more similar fineness ratio to that of *Carcharodon carcharias* and *Ophthalmosaurus*, than to that of *Cymbospondylus* and *Besanosaurus leptorhynchus*. Moreover, *Mixosaurus* possesses smaller hindfins in comparison to other Triassic taxa—with regard to the forefin/hindfin ratio (Table S1), *Mixosaurus* (1.88) falls closer to *Stenopterygius quadriscissus* (1.92) and *Ophthalmosaurus icenicus* (2.3) than to *Besanosaurus leptorhynchus* (1.44) and *Cymbospondylus petrinus* (1.18). Given the phylogenetic position of *Mixosaurus* (e.g., Bindellini et al., 2021; Ji et al., 2016; Jiang et al., 2006; Motani, 1999; this paper), we propose that this taxon, independently and convergently, acquired a combination of morphological adaptations that would later appear in Parvipelvia. These could have granted *Mixosaurus* a more efficient swimming style (in terms of net cost of locomotion) when compared to the swimming style of the coeval, sympatric and larger *Besanosaurus leptorhynchus* and *Cymbospondylus*. Gutarra et al. (2019) effectively demonstrated that large body sizes, as well as non-anguilliform swimming modes, markedly reduced the net cost of locomotion in ichthyosaurs. Therefore, we hypothesize that *Mixosaurus,* being much smaller than *Cymbospondylus* and shastasaur-grade ichthyosaurs and occupying a niche distinct from the two large ichthyosaur taxa from the Besano Formation (Bindellini et al., 2021), achieved a more efficient style at its small body size through the evolution of the aforementioned morphological adaptations. Furthermore, a remarkable (unique among ichthyosaurs) increase in the heights of the vertebral centra in the middle portion of the tail in *Mixosaurus* has been suggested to be an adaptation for a high capability for acceleration (Motani, 1998).

**Fig. 12:**
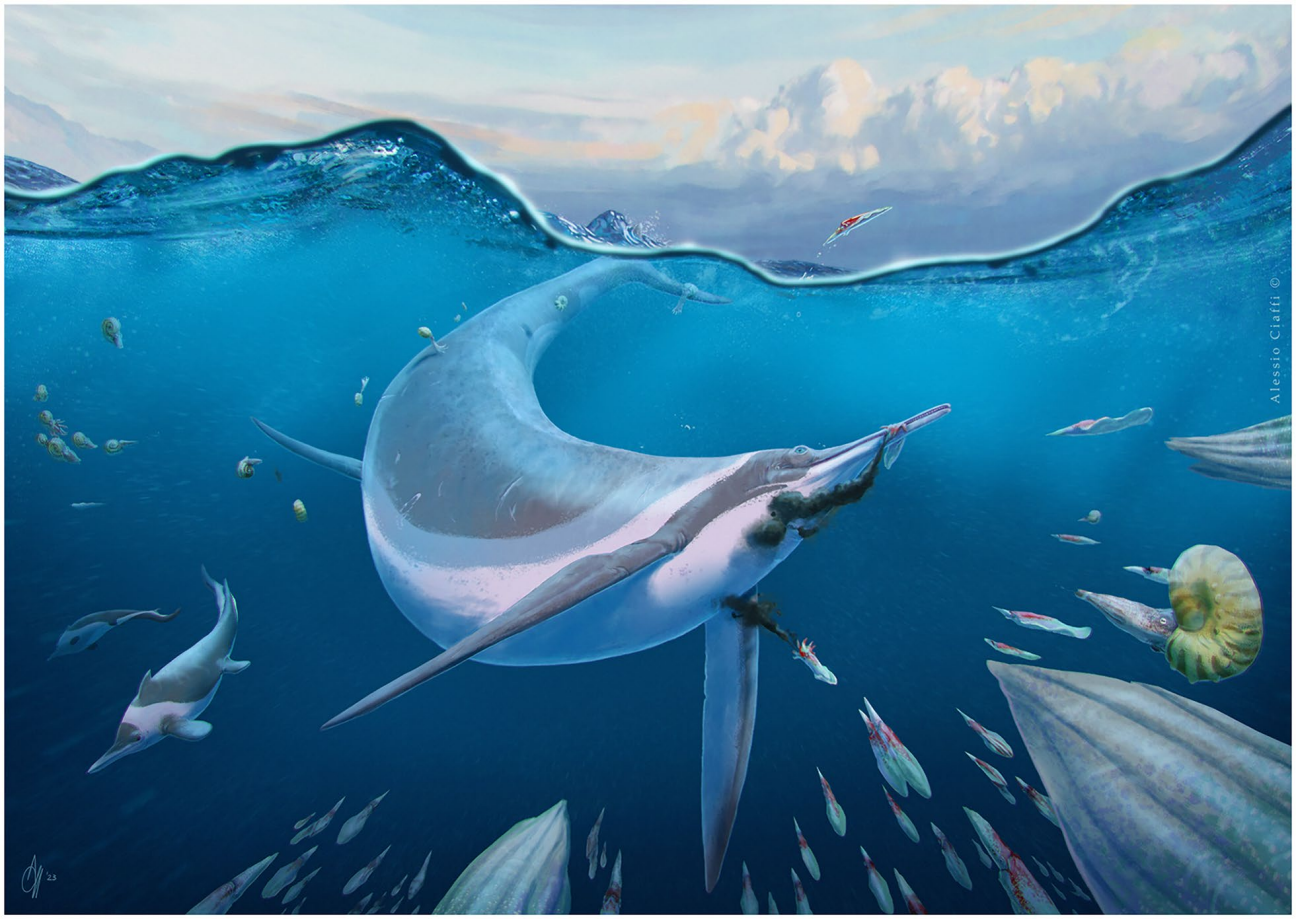
Artistic reconstruction of *Besanosaurus leptorhynchus* (based on BES SC 999) catching a *Phragmoteuthis*. Two individuals of *Mixosaurus* and a shoal of the ammonoid *Ceratites* can be seen in the background to the left of *Besanosaurus leptorhynchus.* Some ammonoids of the genus *Serpianites* are visible swimming around the large ichthyosaur and a single individual of the same ammonoid is depicted in the foreground on the right, along with other specimens of *Phragmoteuthis*. Artwork by Alessio Ciaffi

None of the three ichthyosaurs known from the Besano Formation have been demonstrated to possess a lunate tail fluke. On the other hand, despite the absence of soft tissue in the caudal peak region of the examined specimens of *Besanosaurus leptorhynchus*, the presence of a dorsal lobe of the caudal fin may be inferred, based on the recent discoveries concerning *Mixosaurus* (Renesto et al., 2020). Based on the position of the tailbend, the tail of *Besanosaurus leptorhynchus* likely possessed a markedly heterocercal caudal fluke. A heterocercal tail fluke would have granted greater manoeuverability but would have also resulted in a relatively slower swimming speed (e.g., Thomson & Simanek, 1977; Motani, 2002; Croft et al., 2019). According to Gutarra et al. (2019), this was compensated in *Cymbospondylus* and *Besanosaurus leptorhynchus* by large body size, and in *Mixosaurus* by the adaptations mentioned above. In particular, in *Besanosaurus leptorhynchus,* manoeuvrability could have been enhanced by a highly heterocercal tail and relatively long forefins (as suggested for sharks by e.g., Thomson & Simanek, 1977). Moreover, good manoeuvrability of the body supports the hunting strategy recently hypothesised for *Besanosaurus leptorhynchus*, a longirostrine raptorial snap feeder catching prey by rapid and precise movements of the head and neck (Bindellini et al., 2021; Fig. 12).

Rounded phalanges surrounded by a significant amount of cartilage in the forefins of *Besanosaurus leptorhynchus* would likely result in long and flexible appendages, useful for better manoeuvring of the anterior half of the body. On the other hand, hindfins with subrectangular and packed phalanges might have been stiffer and steadier. Similarly, long and robust hindfins were suggested to enhance manoeuvrability in *Sveltonectes insolitus* (Fischer et al., 2011). Since the main purpose of the dorsal fin is to stabilize the animal against rolling and yawing (e.g., Lingham-Soliar, Lingham-Soliar, 2005; Sanden & Lauder, 2007), aside from enhancing manoeuvrability, long forefins and hindfins in *Besanosaurus* could have compensated for the absence of a dorsal fin (Fig. 12).

## Conclusions

In this work, we described the postcranial anatomy of *Besanosaurus leptorhynchus* based on four specimens, of which BES SC 999 and PIMUZ T 4376 are remarkably complete. This study and the recent publication of the skull material (Bindellini et al., 2021), comprise an extensive and complete revision of this taxon.

*Besanosaurus leptorhynchus* possesses a peculiar body profile intermediate between *Cymbospondylus* and some shastasaur-grade ichthyosaurs. The vertebral count consists of 61 presacral centra, at least two sacral vertebrae, and at least 138 caudal vertebrae. The position and the anatomy of the rib facets show characteristics common with both *Cymbospondylus* and shastasaur-grade ichthyosaurs. The caudal series represents 55% of the length of the whole axial skeleton. The presence of a tailbend that forms an angle of ~ 30° is confirmed and suggests the presence of a heterocercal caudal fluke, more similar to that of shastasaur-grade ichthyosaurs than to that of *Cymbospondylus* and mixosaurids. Remarkably, the pedal phalanges retain reduced shafts, a feature absent in all other shastasaur-grade ichthyosaurs, but also present in *Mixosaurus*. Conversely, forefin phalanges are round as in other shastasaur-grade ichthyosaurs. The rounded phalanges in the forefins were likely surrounded by a significant amount of cartilage, likely making them flexible and useful for better manoeuvering of the anterior half of the body. Such an adaptation is consistent with the ecological niche and hunting strategy previously hypothesised for *Besanosaurus leptorhynchus*. Furthermore, given some similarities in the morphology of the manual phalanges with some modern cetaceans (e.g., orcas, Xie et al., 2020), we suggest that in *Besanosaurus leptorhynchus*, the rounded profile of the forefin elements is achieved by partial loss of perichondral/periosteal bone in the shaft regions. Moreover, individual elements are hypothesised to be set in a (fibro-)cartilaginous rich connective tissue, which would explain their broad spacing.

After updating the phylogenetic scores of *Besanosaurus leptorhynchus* and testing its phylogenetic position we conclude that this taxon represents the earliest diverging member of Merriamosauria. Furthermore, shastasaurs are found to be a grade and not a monophyletic group, corroborating the previous results of Bindellini et al., (2021).

To test the swimming capabilities of *Besanosaurus leptorhynchus* we expanded on the body outline analysis of Motani et al., (1996) by adding 27 ichthyosaurian and fish taxa. This allowed for increasing the representation of the phylogenetic diversity and swimming styles in the dataset. *Besanosaurus leptorhynchus* plots in between anguilliform swimmers, such as *Cymbospondylus*, and shastasaur-grade ichthyosaurs. This result is supported by the anatomy reported in the description and by the recovered phylogenetic position*.* We also propose that *Mixosaurus*, independently and convergently, evolved a combination of adaptations that would later appear in Parvipelvia, such as a relatively rigid trunk, smaller hindfins, and a dorsal fin. These adaptations could have granted *Mixosaurus* a more efficient (in terms of net cost of locomotion) swimming style, when compared to the swimming styles of the larger Triassic ichthyosaurs and were likely related to the small body size of this taxon. Different swimming styles for *Cymbospondylus, Mixosaurus,* and *Besanosaurus leptorhynchus* are supported by the suggested niche partitioning of the three taxa from the Besano basin, as well as by their different anatomy, body proportions, and dimensions.

Previous authors have suggested the possibility that *Besanosaurus* is synonymous with ichthyosaur taxa described from the Triassic of the Svalbard archipelago. After comparing *Besanosaurus leptorhynchus*, *Pessopteryx*, and *Pessosaurus* we conclude that *Besanosaurus leptorhynchus* is not a junior synonym of either of the two taxa.

In summary, this manuscript completes the morphological and taxonomic revision of *Besanosaurus leptorhynchus*, being complementary to the previous work on this ichthyosaur (Bindellini et al., 2021; Dal Sasso & Pinna, 1996). Research on ontogenetic variation and the foetal remains inside the holotype specimen is still ongoing.

## Supplementary Information

Supplementary material 1: Fig. S1 Close-up of the ribcage of BES SC 999, the holotype of *Besanosaurus leptorhynchus* (caudal right quarter; slab 26 following the numbering in Dal Sasso & Pinna, 1996). Scale bar equals 10 cm. Fig. S2 Close-up of the cervico-dorsal region and shoulder girdle of BES SC 999, the holotype of *Besanosaurus leptorhynchus*. Scale bar equals 10 cm. Fig. S3 Close-up of the interclavicle of *Besanosaurus leptorhynchus* (PIMUZ T 4376). Scale bar equals 1 cm. Fig. S4 Strict consensus tree of 14,480 most parsimonious trees of 717 steps (CI 0.361, RI = 0.787). Numbers indicate Bremer support values obtained from parsimony analysis of the phylogenetic matrix in File S1. Fig. S5 Majority rule consensus of 14,480 most parsimonious trees of 717 steps (CI 0.361, RI = 0.787), obtained from parsimony analysis of the phylogenetic matrix in File S1. Note that ‘shastasaurids’ are recovered as a grade at the base of Merriamosauria. Percentages of trees in which particular clades are recovered are reported for each node. Fig. S6 Ichthyosaur and fish silhouettes used as measurement sources for the body shape analysis. Silhouettes of *Salmo*, *Gadus*, *Scomber*, *Thunnus* and *Carcharodon* are based on those available at fisheries.noaa.gov. Fig. S7 High-resolution version of Fig. 11. Full skeletal reconstruction of *Besanosaurus leptorhynchus*. Size and proportions are based on the holotype BES SC 999. Scale bar equals 1 meter. Line drawing by Marco Auditore. Table S1. Ichthyopterygian and fish measurements taken from the silhouettes in Fig. 10. Lengths and heights are given in cm.

## Data Availability

All data generated or analysed during this study are included in this published article and its supplementary information files. The described specimens are available for study at the PIMUZ and MSNM.

## Abbreviations

MSNM: Museo di Storia Naturale di Milano, Milan, Italy
PIMUZ: Department of Paleontology, University of Zurich, Zurich, Switzerland
PMU: Museum of Evolution, Uppsala University, Uppsala, Sweden
