# Supplementary material for: Postcranial anatomy of Besanosaurus leptorhynchus (Reptilia: Ichthyosauria) from the Middle Triassic Besano Formation of Monte San Giorgio (Italy/Switzerland), with implications for reconstructing the swimming styles of Triassic ichthyosaurs
Source: Swiss J Palaeontol. 2024 Sep 10;143(1):32. doi: 10.1186/s13358-024-00330-9 (PMC11384637; doi:10.1186/s13358-024-00330-9)
Supplement: Supplementary file 1 — Supplementary material 1: Fig. S1 Close-up of the ribcage of BES SC 999, the holotype of Besanosaurus leptorhynchus (caudal right quarter; slab 26 following the numbering in Dal Sasso & Pinna, 1996). Scale bar equals 10 cm. Fig. S2 Close-up of the cervico-dorsal region and shoulder girdle of BES SC 999, the holotype of Besanosaurus leptorhynchus. Scale bar equals 10 cm. Fig. S3 Close-up of the interclavicle of Besanosaurus leptorhynchus (PIMUZ T 4376). Scale bar equals 1 cm. Fig. S4 Strict consensus tree of 14,480 most parsimonious trees of 717 steps (CI 0.361, RI = 0.787). Numbers indicate Bremer support values obtained from parsimony analysis of the phylogenetic matrix in File S1. Fig. S5 Majority rule consensus of 14,480 most parsimonious trees of 717 steps (CI 0.361, RI = 0.787), obtained from parsimony analysis of the phylogenetic matrix in File S1. Note that ‘shastasaurids’ are recovered as a grade at the base of Merriamosauria. Percentages of trees in which particular clades are recovered are reported for each node. Fig. S6 Ichthyosaur and fish silhouettes used as measurement sources for the body shape analysis. Silhouettes of Salmo, Gadus, Scomber, Thunnus and Carcharodon are based on those available at fisheries.noaa.gov. Fig. S7 High-resolution version of Fig. 11. Full skeletal reconstruction of Besanosaurus leptorhynchus. Size and proportions are based on the holotype BES SC 999. Scale bar equals 1 meter. Line drawing by Marco Auditore. Table S1. Ichthyopterygian and fish measurements taken from the silhouettes in Fig. 10. Lengths and heights are given in cm. [file 13358_2024_330_MOESM1_ESM.zip › supp material/Table S1.docx]

Table S1. Ichthyopterygian and fish measurements taken from the silhouettes in Fig. 10. Lengths and heights are given in cm.

| **Ref** | **Taxon** | **Prefluke length** | **Max body height (excluding dorsal fin)** | **Tail height** | **Tail length** | **Forefin length** | **Hindfin length** | **Forefin/Hindfin ratio** | **Fineness ratio** | **Tail H/L ratio** |
| --- | --- | --- | --- | --- | --- | --- | --- | --- | --- | --- |
| NOAA fisheries | *Scomber scombrus* | 21,6 | 4,7 | 5,7 | 3,4 | 3,12 | 2,4 | 1,300 | 0,22 | 1,68 |
| NOAA fisheries | *Scomber colias* | 55 | 13 | 15,5 | 9 | 8,5 | 5 | 1,700 | 0,24 | 1,72 |
| NOAA fisheries | *Scomberomorus cavalla* | 147 | 26,5 | 51 | 33 | 18,5 | 7,5 | 2,467 | 0,18 | 1,55 |
| NOAA fisheries | *Scomber japonicus* | 30 | 7 | 8 | 5 | 4 | 3 | 1,333 | 0,23 | 1,60 |
| NOAA fisheries | *Scomberomorus maculatus* | 74,5 | 13 | 23 | 16,5 | 9 | 3,7 | 2,432 | 0,17 | 1,39 |
| NOAA fisheries | *Thunnus thynnus* | 206 | 55 | 73,5 | 25,5 | 40 | 25,4 | 1,575 | 0,27 | 2,88 |
| NOAA fisheries | *Thunnus obesus* | 155 | 46 | 60 | 25 | 42 | 14 | 3,000 | 0,30 | 2,40 |
| NOAA fisheries | *Katsuwonus pelamis* | 71 | 20 | 23 | 9 | 12 | 6 | 2,000 | 0,28 | 2,56 |
| NOAA fisheries | *Thunnus albacares* | 124 | 33,5 | 50,5 | 26 | 33 | 15 | 2,200 | 0,27 | 1,94 |
| NOAA fisheries | *Thunnus alalunga* | 89 | 25 | 31 | 11 | 31 | 7 | 4,429 | 0,28 | 2,82 |
| NOAA fisheries | *Carcharodon carcharias* | 316,4 | 70 | 108 | 80 | 77 | 20,5 | 3,756 | 0,22 | 1,35 |
| Motani et al., 2014 | *Chaohusaurus geishanensis* | 65,2 | 11,5 | 7,8 | 31 | 11,5 | 9,4 | 1,223 | 0,18 | 0,25 |
| Motani et al., 1996 | *Chaohusaurus chaoxianensis* | 50 | 6 | 3,6 | 15,6 | 8 | 7 | 1,143 | 0,12 | 0,23 |
| Motani et al., 1998 | *Utatsusaurus hataii* | 194,7 | 31 | 26,4 | 65,3 | 27,1 | 22,5 | 1,204 | 0,16 | 0,40 |
| Jiang et al., 2008, this paper | *Xinminosaurus catactes* | 198 | 15 | 27,2 | 27 | 26,6 | 23,5 | 1,132 | 0,08 | 1,01 |
| McGowan & Motani, 2003 | *Cymbospondylus petrinus* | 702 | 90 | 85 | 218 | 108,8 | 91,7 | 1,186 | 0,13 | 0,39 |
| Renesto et al., 2020 | *Mixosaurus cornalianus* | 146,1 | 31,8 | 21,8 | 47,2 | 27,7 | 14,7 | 1,884 | 0,22 | 0,46 |
| This paper | *Besanosaurus leptorhynchus* | 370 | 49 | 80 | 98 | 77,5 | 54 | 1,435 | 0,13 | 0,82 |
| Motani, 2008 + Ji et al., 2013 + Sander et al., 2011 | *Gualingsaurus liangae* | 392 | 65 | 75 | 118 | 63 | 65 | 0,969 | 0,17 | 0,64 |
| Shang & Li, 2009 | *Guizhouichthyosaurus tangae* | 487 | 69,5 | 87 | 97 | 120 | 95 | 1,263 | 0,14 | 0,90 |
| McGowan & Motani, 2003 | *Californosaurus perrini* | 215 | 41,5 | 48 | 36 | 35 | 27 | 1,296 | 0,19 | 1,33 |
| McGowan & Motani, 2003 | *Temnodontosaurus platyodon* | 604 | 105 | 165 | 72 | 95,7 | 75 | 1,276 | 0,17 | 2,29 |
| McGowan & Motani, 2003 | *Eurhinosaurus longirostris* | 468 | 60 | 150 | 88 | 102 | 84 | 1,214 | 0,13 | 1,70 |
| Maxwell & Cortés, 2020 | *Hauffiopteryx typicus* | 150,7 | 28 | 38,5 | 23,2 | 24,5 | 9,4 | 2,606 | 0,19 | 1,66 |
| Motani et al., 2014 | *Stenopterygius quadriscissus* | 195,3 | 42 | 54,3 | 34,7 | 33,5 | 12 | 2,792 | 0,22 | 1,56 |
| Motani et al., 2014 | *Stenopterygius quadriscissus* (new born) | 54 | 9,5 | 8 | 9 | 6,7 | 3,8 | 1,763 | 0,18 | 0,89 |
| McGowan & Motani, 2003 | *Ophthalmosaurus icenicus* | 387 | 94 | 142,1 | 53,5 | 69,6 | 30,3 | 2,297 | 0,24 | 2,66 |
