# Supplementary figures and images for: Postcranial anatomy of Besanosaurus leptorhynchus (Reptilia: Ichthyosauria) from the Middle Triassic Besano Formation of Monte San Giorgio (Italy/Switzerland), with implications for reconstructing the swimming styles of Triassic ichthyosaurs
Source: Swiss J Palaeontol. 2024 Sep 10;143(1):32. doi: 10.1186/s13358-024-00330-9 (PMC11384637; doi:10.1186/s13358-024-00330-9)

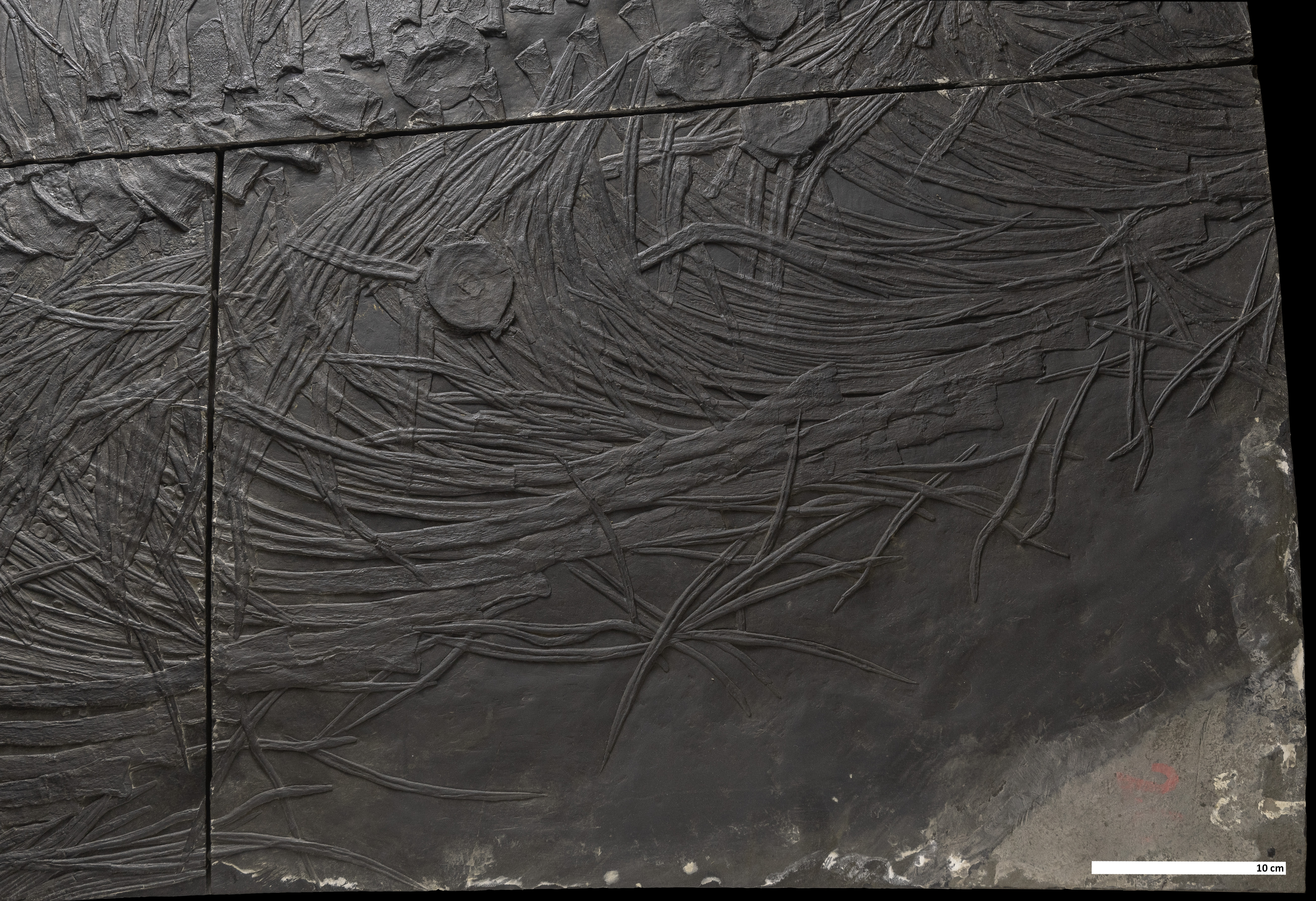

Supplement: Supplementary file 1 — Supplementary material 1: Fig. S1 Close-up of the ribcage of BES SC 999, the holotype of Besanosaurus leptorhynchus (caudal right quarter; slab 26 following the numbering in Dal Sasso & Pinna, 1996). Scale bar equals 10 cm. Fig. S2 Close-up of the cervico-dorsal region and shoulder girdle of BES SC 999, the holotype of Besanosaurus leptorhynchus. Scale bar equals 10 cm. Fig. S3 Close-up of the interclavicle of Besanosaurus leptorhynchus (PIMUZ T 4376). Scale bar equals 1 cm. Fig. S4 Strict consensus tree of 14,480 most parsimonious trees of 717 steps (CI 0.361, RI = 0.787). Numbers indicate Bremer support values obtained from parsimony analysis of the phylogenetic matrix in File S1. Fig. S5 Majority rule consensus of 14,480 most parsimonious trees of 717 steps (CI 0.361, RI = 0.787), obtained from parsimony analysis of the phylogenetic matrix in File S1. Note that ‘shastasaurids’ are recovered as a grade at the base of Merriamosauria. Percentages of trees in which particular clades are recovered are reported for each node. Fig. S6 Ichthyosaur and fish silhouettes used as measurement sources for the body shape analysis. Silhouettes of Salmo, Gadus, Scomber, Thunnus and Carcharodon are based on those available at fisheries.noaa.gov. Fig. S7 High-resolution version of Fig. 11. Full skeletal reconstruction of Besanosaurus leptorhynchus. Size and proportions are based on the holotype BES SC 999. Scale bar equals 1 meter. Line drawing by Marco Auditore. Table S1. Ichthyopterygian and fish measurements taken from the silhouettes in Fig. 10. Lengths and heights are given in cm. [file 13358_2024_330_MOESM1_ESM.zip › supp material/Fig. S1R3.jpg]

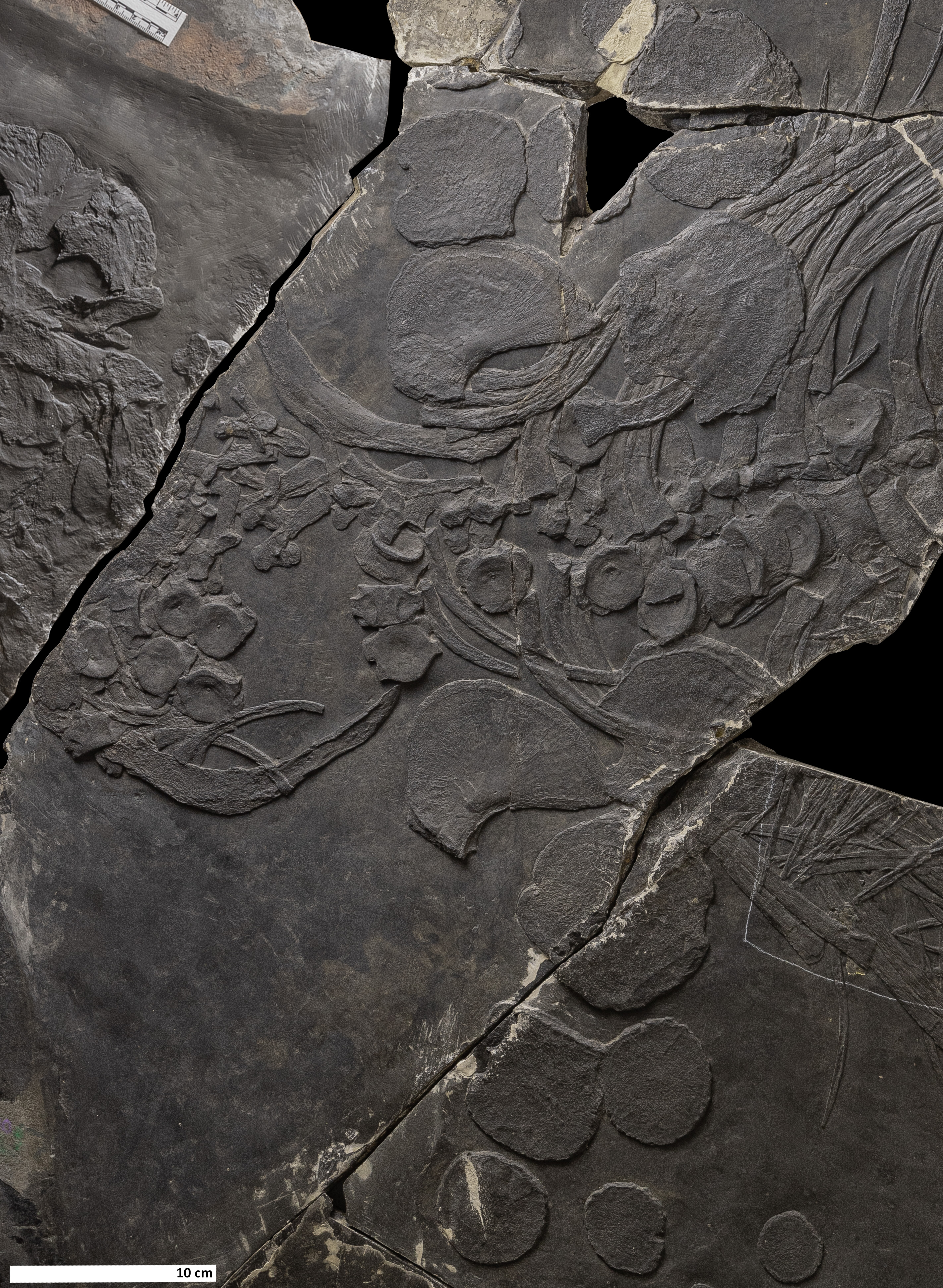

Supplement: Supplementary file 1 — Supplementary material 1: Fig. S1 Close-up of the ribcage of BES SC 999, the holotype of Besanosaurus leptorhynchus (caudal right quarter; slab 26 following the numbering in Dal Sasso & Pinna, 1996). Scale bar equals 10 cm. Fig. S2 Close-up of the cervico-dorsal region and shoulder girdle of BES SC 999, the holotype of Besanosaurus leptorhynchus. Scale bar equals 10 cm. Fig. S3 Close-up of the interclavicle of Besanosaurus leptorhynchus (PIMUZ T 4376). Scale bar equals 1 cm. Fig. S4 Strict consensus tree of 14,480 most parsimonious trees of 717 steps (CI 0.361, RI = 0.787). Numbers indicate Bremer support values obtained from parsimony analysis of the phylogenetic matrix in File S1. Fig. S5 Majority rule consensus of 14,480 most parsimonious trees of 717 steps (CI 0.361, RI = 0.787), obtained from parsimony analysis of the phylogenetic matrix in File S1. Note that ‘shastasaurids’ are recovered as a grade at the base of Merriamosauria. Percentages of trees in which particular clades are recovered are reported for each node. Fig. S6 Ichthyosaur and fish silhouettes used as measurement sources for the body shape analysis. Silhouettes of Salmo, Gadus, Scomber, Thunnus and Carcharodon are based on those available at fisheries.noaa.gov. Fig. S7 High-resolution version of Fig. 11. Full skeletal reconstruction of Besanosaurus leptorhynchus. Size and proportions are based on the holotype BES SC 999. Scale bar equals 1 meter. Line drawing by Marco Auditore. Table S1. Ichthyopterygian and fish measurements taken from the silhouettes in Fig. 10. Lengths and heights are given in cm. [file 13358_2024_330_MOESM1_ESM.zip › supp material/Fig. S2R3.jpg]

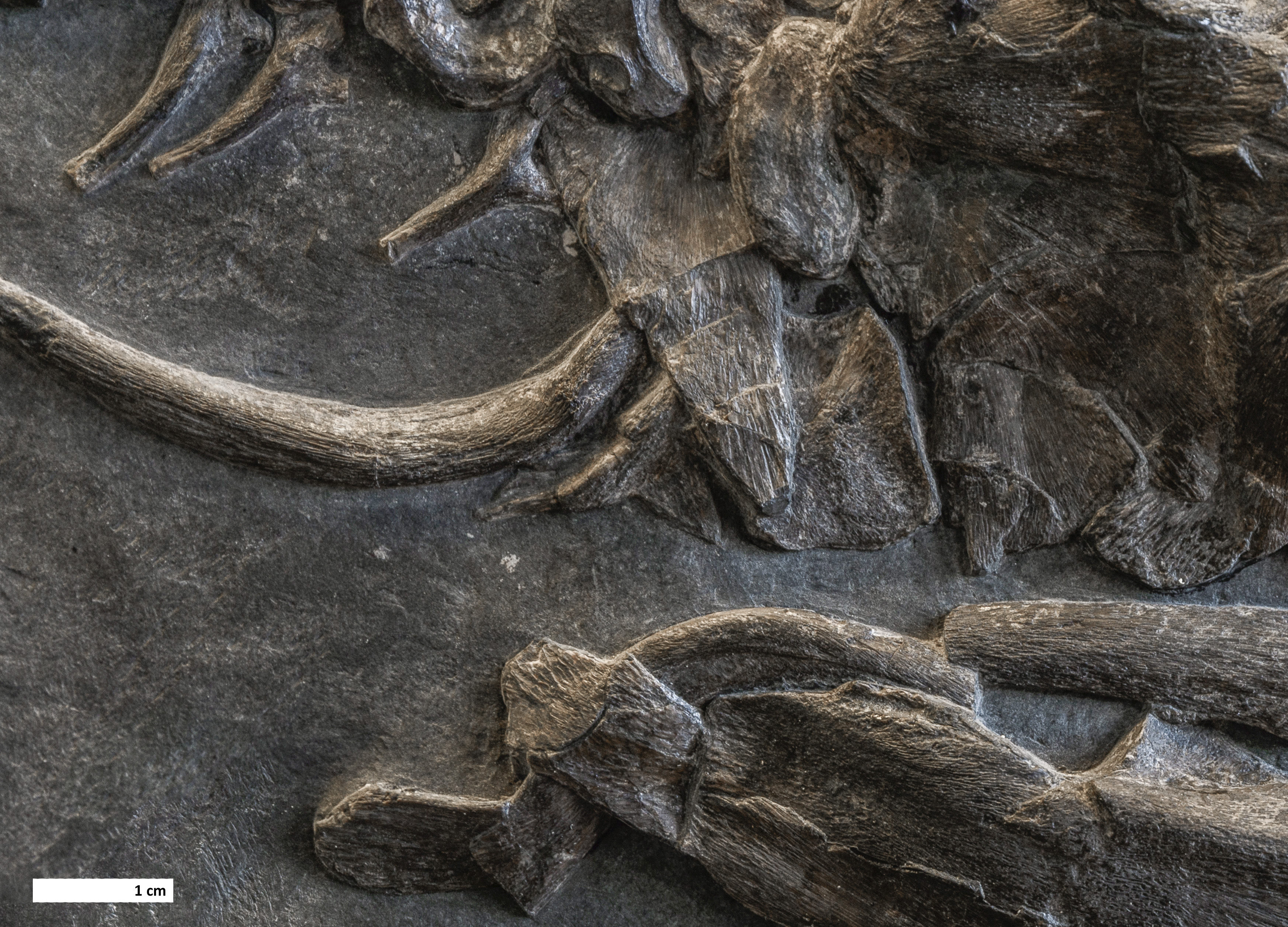

Supplement: Supplementary file 1 — Supplementary material 1: Fig. S1 Close-up of the ribcage of BES SC 999, the holotype of Besanosaurus leptorhynchus (caudal right quarter; slab 26 following the numbering in Dal Sasso & Pinna, 1996). Scale bar equals 10 cm. Fig. S2 Close-up of the cervico-dorsal region and shoulder girdle of BES SC 999, the holotype of Besanosaurus leptorhynchus. Scale bar equals 10 cm. Fig. S3 Close-up of the interclavicle of Besanosaurus leptorhynchus (PIMUZ T 4376). Scale bar equals 1 cm. Fig. S4 Strict consensus tree of 14,480 most parsimonious trees of 717 steps (CI 0.361, RI = 0.787). Numbers indicate Bremer support values obtained from parsimony analysis of the phylogenetic matrix in File S1. Fig. S5 Majority rule consensus of 14,480 most parsimonious trees of 717 steps (CI 0.361, RI = 0.787), obtained from parsimony analysis of the phylogenetic matrix in File S1. Note that ‘shastasaurids’ are recovered as a grade at the base of Merriamosauria. Percentages of trees in which particular clades are recovered are reported for each node. Fig. S6 Ichthyosaur and fish silhouettes used as measurement sources for the body shape analysis. Silhouettes of Salmo, Gadus, Scomber, Thunnus and Carcharodon are based on those available at fisheries.noaa.gov. Fig. S7 High-resolution version of Fig. 11. Full skeletal reconstruction of Besanosaurus leptorhynchus. Size and proportions are based on the holotype BES SC 999. Scale bar equals 1 meter. Line drawing by Marco Auditore. Table S1. Ichthyopterygian and fish measurements taken from the silhouettes in Fig. 10. Lengths and heights are given in cm. [file 13358_2024_330_MOESM1_ESM.zip › supp material/Fig. S3R3.jpg]

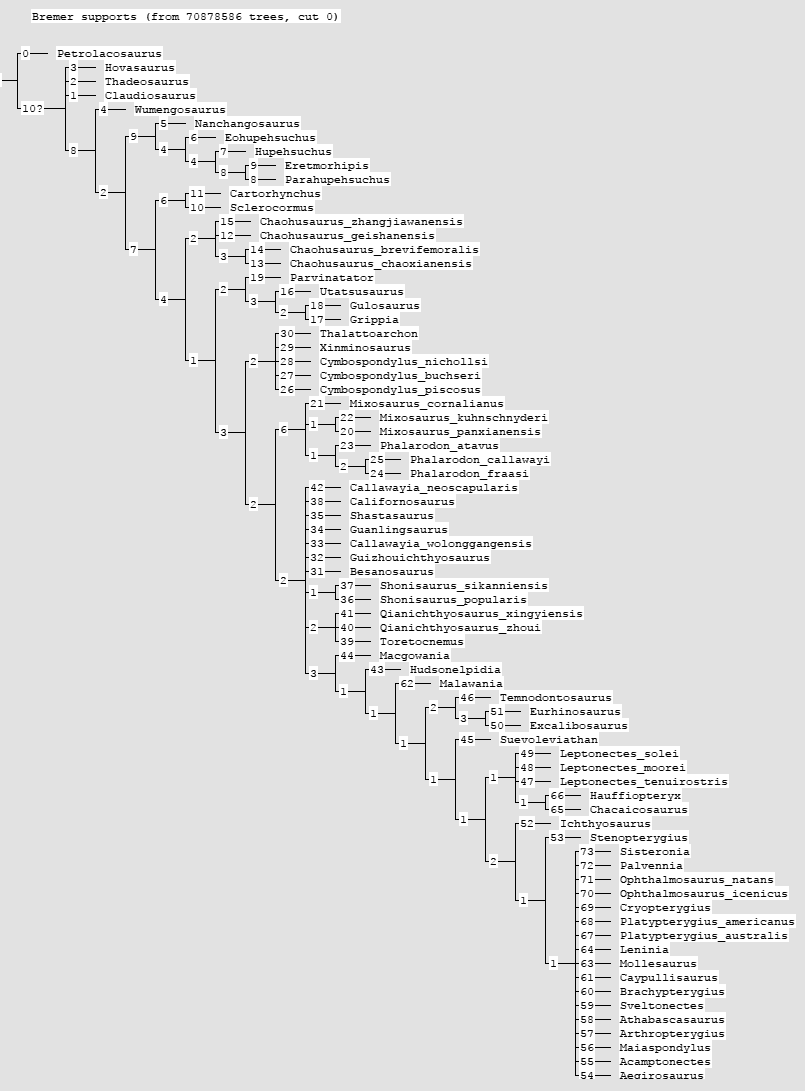

Supplement: Supplementary file 1 — Supplementary material 1: Fig. S1 Close-up of the ribcage of BES SC 999, the holotype of Besanosaurus leptorhynchus (caudal right quarter; slab 26 following the numbering in Dal Sasso & Pinna, 1996). Scale bar equals 10 cm. Fig. S2 Close-up of the cervico-dorsal region and shoulder girdle of BES SC 999, the holotype of Besanosaurus leptorhynchus. Scale bar equals 10 cm. Fig. S3 Close-up of the interclavicle of Besanosaurus leptorhynchus (PIMUZ T 4376). Scale bar equals 1 cm. Fig. S4 Strict consensus tree of 14,480 most parsimonious trees of 717 steps (CI 0.361, RI = 0.787). Numbers indicate Bremer support values obtained from parsimony analysis of the phylogenetic matrix in File S1. Fig. S5 Majority rule consensus of 14,480 most parsimonious trees of 717 steps (CI 0.361, RI = 0.787), obtained from parsimony analysis of the phylogenetic matrix in File S1. Note that ‘shastasaurids’ are recovered as a grade at the base of Merriamosauria. Percentages of trees in which particular clades are recovered are reported for each node. Fig. S6 Ichthyosaur and fish silhouettes used as measurement sources for the body shape analysis. Silhouettes of Salmo, Gadus, Scomber, Thunnus and Carcharodon are based on those available at fisheries.noaa.gov. Fig. S7 High-resolution version of Fig. 11. Full skeletal reconstruction of Besanosaurus leptorhynchus. Size and proportions are based on the holotype BES SC 999. Scale bar equals 1 meter. Line drawing by Marco Auditore. Table S1. Ichthyopterygian and fish measurements taken from the silhouettes in Fig. 10. Lengths and heights are given in cm. [file 13358_2024_330_MOESM1_ESM.zip › supp material/Fig. S4 strict consensus treeR3.png]

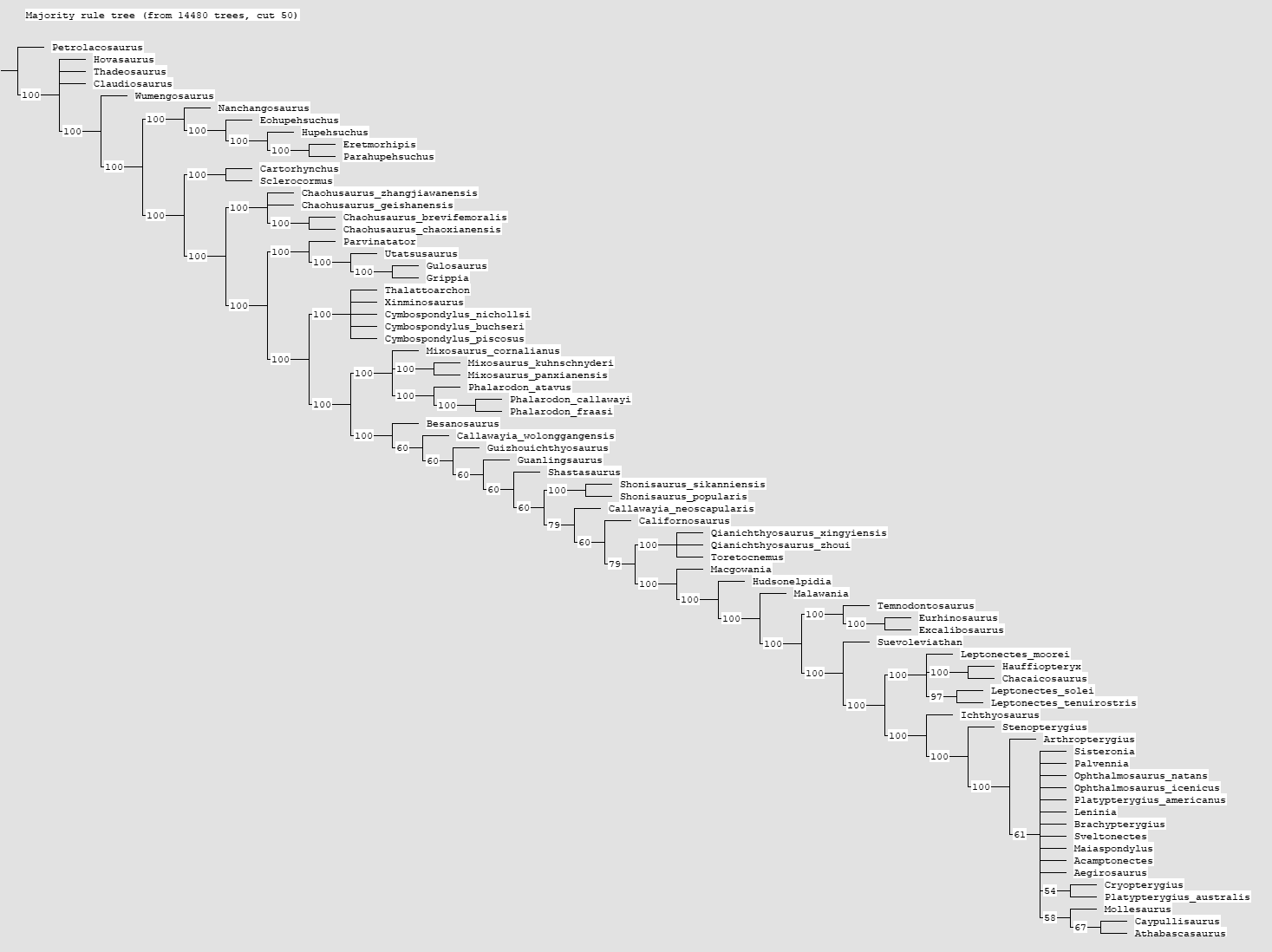

Supplement: Supplementary file 1 — Supplementary material 1: Fig. S1 Close-up of the ribcage of BES SC 999, the holotype of Besanosaurus leptorhynchus (caudal right quarter; slab 26 following the numbering in Dal Sasso & Pinna, 1996). Scale bar equals 10 cm. Fig. S2 Close-up of the cervico-dorsal region and shoulder girdle of BES SC 999, the holotype of Besanosaurus leptorhynchus. Scale bar equals 10 cm. Fig. S3 Close-up of the interclavicle of Besanosaurus leptorhynchus (PIMUZ T 4376). Scale bar equals 1 cm. Fig. S4 Strict consensus tree of 14,480 most parsimonious trees of 717 steps (CI 0.361, RI = 0.787). Numbers indicate Bremer support values obtained from parsimony analysis of the phylogenetic matrix in File S1. Fig. S5 Majority rule consensus of 14,480 most parsimonious trees of 717 steps (CI 0.361, RI = 0.787), obtained from parsimony analysis of the phylogenetic matrix in File S1. Note that ‘shastasaurids’ are recovered as a grade at the base of Merriamosauria. Percentages of trees in which particular clades are recovered are reported for each node. Fig. S6 Ichthyosaur and fish silhouettes used as measurement sources for the body shape analysis. Silhouettes of Salmo, Gadus, Scomber, Thunnus and Carcharodon are based on those available at fisheries.noaa.gov. Fig. S7 High-resolution version of Fig. 11. Full skeletal reconstruction of Besanosaurus leptorhynchus. Size and proportions are based on the holotype BES SC 999. Scale bar equals 1 meter. Line drawing by Marco Auditore. Table S1. Ichthyopterygian and fish measurements taken from the silhouettes in Fig. 10. Lengths and heights are given in cm. [file 13358_2024_330_MOESM1_ESM.zip › supp material/Fig. S5 majority rule consensus treeR3.png]
